# Myeloid PFKFB3-mediated glycolysis promotes kidney fibrosis

**DOI:** 10.3389/fimmu.2023.1259434

**Published:** 2023-11-16

**Authors:** Qiuhua Yang, Emily Huo, Yongfeng Cai, Zhidan Zhang, Charles Dong, John M. Asara, Huidong Shi, Qingqing Wei

**Affiliations:** ^1^ Department of Cellular Biology and Anatomy, Medical College of Georgia, Augusta University, Augusta, GA, United States; ^2^ Augusta Preparatory Day School, Martinez, GA, United States; ^3^ Dental College of Georgia, Augusta University, Augusta, GA, United States; ^4^ Division of Signal Transduction, Beth Israel Deaconess Medical Center and Department of Medicine, Harvard Medical School, Boston, MA, United States; ^5^ Department of Biochemistry and Molecular Biology, Medical College of Georgia, Augusta University, Augusta, GA, United States; ^6^ Georgia Cancer Center, Medical College of Georgia, Augusta University, Augusta, GA, United States

**Keywords:** macrophage, glycolysis, renal fibrosis, PFKFB3, inflammation

## Abstract

Excessive renal fibrosis is a common pathology in progressive chronic kidney diseases. Inflammatory injury and aberrant repair processes contribute to the development of kidney fibrosis. Myeloid cells, particularly monocytes/macrophages, play a crucial role in kidney fibrosis by releasing their proinflammatory cytokines and extracellular matrix components such as collagen and fibronectin into the microenvironment of the injured kidney. Numerous signaling pathways have been identified in relation to these activities. However, the involvement of metabolic pathways in myeloid cell functions during the development of renal fibrosis remains understudied. In our study, we initially reanalyzed single-cell RNA sequencing data of renal myeloid cells from Dr. Denby’s group and observed an increased gene expression in glycolytic pathway in myeloid cells that are critical for renal inflammation and fibrosis. To investigate the role of myeloid glycolysis in renal fibrosis, we utilized a model of unilateral ureteral obstruction in mice deficient of *Pfkfb3*, an activator of glycolysis, in myeloid cells *(Pfkfb3* ^ΔMϕ^ *)* and their wild type littermates (*Pfkfb3* ^WT^). We observed a significant reduction in fibrosis in the obstructive kidneys of *Pfkfb3* ^ΔMϕ^ mice compared to *Pfkfb3* ^WT^ mice. This was accompanied by a substantial decrease in macrophage infiltration, as well as a decrease of M1 and M2 macrophages and a suppression of macrophage to obtain myofibroblast phenotype in the obstructive kidneys of *Pfkfb3* ^ΔMϕ^ mice. Mechanistic studies indicate that glycolytic metabolites stabilize HIF1α, leading to alterations in macrophage phenotype that contribute to renal fibrosis. In conclusion, our study implicates that targeting myeloid glycolysis represents a novel approach to inhibit renal fibrosis.

## Introduction

Fibrosis is a pathological condition commonly observed in advanced stages of organ disease, such as chronic kidney disease (CKD) (1). The progression of renal fibrosis relies heavily on the activation of myofibroblasts, a specialized type of fibroblast characterized by the expression of alpha-smooth muscle actin (α-SMA). These myofibroblasts play a pivotal role in renal fibrosis by producing and secreting excessive extracellular matrix proteins such as fibronectin and collagens (2) and other components to affect renal function (3). Various cell types have been identified as potential sources of myofibroblasts involved in the development of renal fibrosis including resident fibroblasts, hematopoietic cells, and pericytes (4–11).

Extensive research has elucidated the significant involvement of myeloid cells, particularly monocytes/macrophages, in the development of renal fibrosis (12–17). During the early stage of renal injury, upon the stimulation of various inflammatory cytokines (18, 19), circulating monocytes are recruited to the injured kidney and undergo differentiation into M1 macrophages. These M1 macrophages not only proliferate locally but also have the ability to differentiate to be M2 macrophages in response to the changes in the microenvironment (20). Additionally, a subset of M2 macrophages can further undergo a phenotype change, acquiring some characteristics of myofibroblasts, which has been described as macrophage-myofibroblast transition (MMT) in some recent studies (11, 14, 21–23). Inhibition of monocyte infiltration into the kidney (24–26), attenuation of renal injury caused by M1 macrophages (27) (28), as well as modulation of fibrotic activity associated with M2 and MMT processes (14, 29), have all been shown to inhibit the progression of renal fibrosis effectively.

Metabolic reprogramming, particularly the shift toward glycolysis, plays a critical role in various pathological cellular procedures such as tumor cell growth and metastasis (30, 31), drug resistant epilepsy (32), and the function and polarization of myeloid cells (33). One important enzyme involved in this process is 6-phosphofructo-2-kinase/fructose-2,6-bisphosphatase 3 (PFKFB3), which catalyzes the synthesis of fructose-2, 6-bisphosphate (F-2,6-P2). F-2,6-P2 serves as a potent allosteric activator of 6-phosphofructo-1-kinase (PFK-1), one of key rate-limiting enzymes in glycolysis (34). Previous studies utilizing mice deficient in myeloid Pfkfb3 have demonstrated its role in suppressing ocular angiogenesis (35, 36), pulmonary hypertension (37), and atherosclerosis (38). Metabolic shifting to glycolysis has been noted in renal repair and fibrosis development with renal acidosis developed after the accumulation of lactate (39, 40). In studies from our lab and other research groups, glycolysis has emerged as a notable contributor to renal fibrosis with divergent effect on different renal cells, and glycolysis inhibitors can reduce the macrophage infiltration in renal fibrosis (41–43). However, the effect of glycolysis on macrophage differentiation and the significance of myeloid PFKFB3-mediated glycolysis in the development of renal fibrosis has yet to be fully elucidated. In this study, we employed a combination of *in vitro* and *in vivo* approaches to investigate the role of myeloid PFKFB3 in myeloid cell infiltration to kidney, renal inflammation, M2 macrophage differentiation, and ultimately the development of renal fibrosis in the unilateral ureteral obstruction (UUO) model.

## Method

### Mouse generation

All animal care and experimental procedures were conducted in compliance with the National Institutes of Health Guide for the Care and Use of Laboratory Animals. The protocol (Animal protocol # 2011-0401 and # 2018-0971) followed was approved by the Institutional Animal Care and Use Committee at Augusta University. Prior to, during, and after the experiment, welfare assessments, measurements, and interventions were performed. The mice were housed under controlled environmental conditions, including a temperature range of 21-25 °C, humidity between 40-60%, and a 12-hour light/dark cycle. All animals had ad libitum access to food (Teklad global 18% protein rodent diet; 2918-060622M, Envigo, Madison, WI, USA) and water. Floxed *Pfkfb3* (*Pfkfb3* ^flox/flox^) mice were generated by Xenogen Biosciences Corporation (Cranbury, NJ, USA) (44). To achieve cell-specific deficiency of *Pfkfb3* in macrophages, *Pfkfb3* ^flox/flox^ mice were crossbred with Lysm-Cre transgenic mice (The Jackson Laboratory, stock no. 004781, Bar Harbor, ME, USA) to generate *Pfkfb3* ^flox/flox^ Lysm-Cre (*Pfkfb3* ^ΔMφ^) mice, with *Pfkfb3* ^WT/WT^ Lysm-Cre (*Pfkfb3* ^WT^) mice used as wild type control. All mice were genotyped by PCR amplification of tail-clip samples (**Supplementary Table 1**).

### Unilateral ureteral obstruction mouse model

As described previously (41), Adult male mice weighing between 20-25 grams were used for this study. Under anesthesia induced by i.p. injection of ketamine (100 mg/kg) and xylazine (10 mg/kg), a midline abdominal incision was made to expose the left ureter. A double-ligature technique was utilized to create a complete obstruction by tying a suture around the left ureter at two distinct points, with a 1-mm interval between the ligatures. The ureter was then gently compressed between the ligatures and carefully examined to ensure complete occlusion. The mice were allowed to recover under controlled temperature and humidity conditions. At designated time points, the mice were euthanized using i.p. injection of ketamine (100 mg/kg) and xylazine (10 mg/kg) to collect kidney samples for analysis. The kidneys were carefully dissected and processed for histological examination and gene expression studies. The contralateral kidneys without ligation were used as control.

### Culture of bone marrow-derived macrophages

After euthanizing the mice, femurs and tibias were collected and transected. The bone marrow cells were flushed out from the femurs and tibias. These cells were then passed through a 70 μm cell strainer and centrifuged at 1000 x g for 3 minutes to obtain a single-cell suspension. The collected cells were plated onto a 10 cm^2^ dish and allowed to incubate for 6-8 hours to discard the attached cells. The remaining suspension cells were collected and reseeded into a 6-well plate at a density of 2 x 10^6^ cells/mL. Cells were cultured in RPMI 1640 medium (SH30027.01, Cytiva, Marlborough, MA, USA) supplemented with 10% FBS (F4135, Sigma-Aldrich, St. Louis, MO, USA), 10 ng/mL MCSF (315-02, PeproTech, Cranbury, NJ, USA) and 1% Antibiotic-Antimycotic (15240062, Thermo Scientific, Grand Island, NY, USA) in a humidified incubator with 5% CO2 at 37°C for 7 days to induce macrophage differentiation as described before (45). After 7 days, the differentiated cells were cultured in RPMI 1640 medium supplemented with 1% FBS, 1% Antibiotic-Antimycotic, and 10 ng/mL MCSF. Subsequently, the cells were treated with or without 10 ng/mL mouse TGFβ1 (7666-MB-005, R&D SYSTEMS, Minneapolis, MN, USA) to induce macrophage differentiation for 5 days.

### Real-time PCR

To extract total RNA from cells or tissues, the Trizol Reagent (15596018, Invitrogen, Grand Island, NY) was employed following the manufacturer’s protocol, as previously described (46). For cDNA synthesis, the iScriptTM cDNA synthesis kit (1708891, Bio-Rad Hercules, CA, USA) was utilized. RT-qPCR was performed on a StepOne Plus System (Applied Biosystems, Grand Island, NY) using the Power SYBR Green Master Mix (1725122, Bio-Rad Hercules). The relative gene expression was quantified using the efficiency-corrected 2^−△△CT^ method, with 18S ribosomal RNA serving as the internal control. The obtained data are presented as fold change relative to the control groups. **Supplementary Table 2** includes the list of gene-specific primers employed in our study.

### Western blot analysis

Protein extracts were obtained from kidney tissues and cells by lysing them in RIPA buffer supplemented with protease inhibitor cocktails (05892970001, Roche, SC, USA). The kidney tissues were ground and then lysed to prepare the tissue lysates. The extracts were centrifuged at 12,000 rpm for 10 minutes, and the resulting supernatant was collected. The protein concentration in the supernatant was determined using the BCA assay. Subsequently, the samples were subjected to SDS-PAGE and transferred onto nitrocellulose membrane. After blocking with 5% skim milk for one hour, the blots were probed with the following primary antibodies: PFKFB3 (diluted 1:1000, ab181861, abcam), ACTA2 (diluted 1:1000, sc-56499, Santa Cruz), Vimentin (diluted 1:1000, CST5741, Cell Signaling Technology), Collagen I (diluted 1:1000, NB600-408, NOVUS), Collagen IV (diluted 1:1000, ab6586, abcam), Fibronectin (diluted 1:1000, ab2413, abcam), Cyclophilin B (diluted 1:1000, CST43603, Cell Signaling Technology), HIF1A (diluted 1:500, AF1935-SP, R&D systems). Anti-β-actin (diluted 1:1000, sc47776, Santa Cruz) and GAPDH (diluted 1:1000, 2118, CST) were used as loading controls. The antibodies used are provided in **Supplementary Table 3**. The western blots were quantified with a method described previously (47).

### Immunofluorescence

Cells were cultured in 8-Chambered Cell Culture Slides (08-774-26, Fisher Scientific). Frozen or paraffin blocks were sectioned at 7 μm thickness using a Microm cryostat or paraffin microtome. For cells or frozen sections, a PBS wash was performed, followed by fixation with 4% paraformaldehyde for 15 minutes. Subsequently, permeabilization was achieved by treating the cells or slides with 0.5% Triton X-100 for 20 minutes at room temperature. In the case of paraffin sections, slides were subjected to deparaffinization and rehydration before permeabilization. For antigen retrieval, slides were heated in citrate acid buffer (10 mM, pH 6.0) by microwaving at 98°C for 10 minutes. Following these steps, cells or sections were blocked with 10% normal goat serum (50062Z, Thermo Scientific) for 1 hour at room temperature. Primary antibodies against PFKFB3, ACTA2, F4/80, Arg1, IL1β, or COL1 were then added and incubated overnight at 4°C in a humidified chamber. After washing, samples were incubated with Alexa Fluor 488, 594, or 647-conjugated secondary antibodies for 1 hour at room temperature. Nuclei were counterstained with DAPI for 10 minutes at room temperature. The imaging process was carried out using an inverted confocal microscope (Zeiss 780, Carl Zeiss). The images were quantified with a method described previously (35). Four images/section/kidney were used for statistic purpose. The primary antibodies used in our study can be found in **Supplementary Table 3**.

### Immunohistochemistry

Paraffin-embedded kidneys were sectioned and underwent deparaffinization using xylene, followed by rehydration with a gradient of ethanol and water solutions. The activity of endogenous peroxidase in the tissue was eliminated by a treatment with methanol containing 30% H_2_O_2_ for 30 minutes at room temperature. Antigen retrieval was performed by exposing the sections to 10 mM sodium citrate buffer (pH 6.0) at 98°C for 10 minutes, followed by blocking with avidin blocking solution for 1 hour at room temperature. The antibodies were mixed with biotin blocking solution as per the manufacturer’s instructions and applied to the slides, which were then incubated overnight at 4°C. Subsequently, the tissue sections were treated with a biotinylated secondary antibody for 1 hour at room temperature, followed by incubation with ABC solution (PK-6100, Vector Laboratories) for 30 minutes at room temperature. The antibody was visualized using the peroxidase substrate 3, 3’-diaminobenzidine (3468, Dako, Santa Clara, CA, USA). Hematoxylin I (GHS116, Sigma) was used for counterstaining of the kidney sections. Finally, the slides were dehydrated and mounted using a xylene-based mounting medium (8312-4, Richard-Allan Scientific). The image data was quantified with the method described previously (48), using Image J Software (National Institutes of Health, USA, http://imagej.nih.gov/ij). Four images/section/kidney were used for statistic purpose. The primary antibodies used in our study can be found in **Supplementary Table 3**.

### Histological staining

We performed H & E staining on 7 μm sections of kidney with hematoxylin (22050111, Thermo Scientific) and eosin (220501110, Thermo Scientific) following the manufacturer’s standard protocol. Collagen deposition was visualized using Masson’s Trichrome staining kit (25088-1, Polysciences, PA, USA) and Sirius Red staining kit (ab150681, Abcam, CA, USA) according to the manufacturer’s instructions. Four images of were randomly taken in cortex/outer medulla region per section for each kidney, captured with a Keyence Microscope BZ-X800, and the percentage of collagen deposition area was quantified with Image J Software (National Institutes of Health, USA, http://imagej.nih.gov/ij).

### Metabolomics assay

In the experiment, biological triplicate 10 cm^2^ dishes were utilized to cultivate BMDMs in the presence of complete cell medium. Following the specific treatment, metabolites were extracted using 1 mL of ice-cold 80% methanol on dry ice. Subsequently, the samples underwent centrifugation at 14000 rpm for a duration of 5 minutes. To ensure thorough extraction, the cell pellet was subjected to an additional step involving the use of 0.5 mL of 80% methanol. For accurate protein quantitation, the cell pellet was dissolved in an 8 M urea solution. To facilitate convenient shipment or storage, the supernatant obtained from the metabolite extraction was desiccated into a pellet using a SpeedVac from Eppendorf (Hamburg, Germany), employing a heat-free technique. When it was time for analysis, the dried pellets were re-suspended in 20 μL of HPLC grade water in preparation for mass spectrometry as described before (49). A volume of 5-7 μL of the resulting resuspension was injected and subjected to analysis using a cutting-edge hybrid 6500 QTRAP triple quadrupole mass spectrometer from AB/SCIEX (MA, USA), which was coupled to a Prominence UFLC HPLC system from Shimadzu (Kyoto, Japan). The analysis was carried out through selected reaction monitoring (SRM), targeting a comprehensive set of 298 endogenous water-soluble metabolites, enabling a thorough examination of the steady-state characteristics of the samples. The original data has been uploaded to MetaboLights (MTBLS8278) for public data sharing.

### Measurement of cytokines

The levels of mouse cytokines TNFα and IL1β were measured with ELISA kits (MTA00B and MLB00C, R&D, Minneapolis, MN, USA) according to the manufacturer’s instructions.

### Statistical analysis

Statistical analysis was conducted using GraphPad Prism Software (Version 9.0, RRID: SCR_000306). The significance of differences between two groups was evaluated using the unpaired Student’s t-test or unpaired two-tailed Student’s t-test with Welch’s correction (with unequal variances). Multiple comparisons were performed through one-way ANOVA followed by Bonferroni *post hoc* test. All results are presented as mean ± SEM. A significance level of P < 0.05 was considered statistically significant. To calculate the Z-score, we used the formula below: Z-score = (x - μ)/σ, x is the value of the data point, μ is the mean of the sample or data set, σ is the standard deviation. Each biological experiment was repeated a minimum of three times, utilizing independent cell cultures or individual animals as biological replications.

## Results

### Increased glycolytic gene expression in myeloid cells infiltrating in the UUO kidney

**Figure 1:**
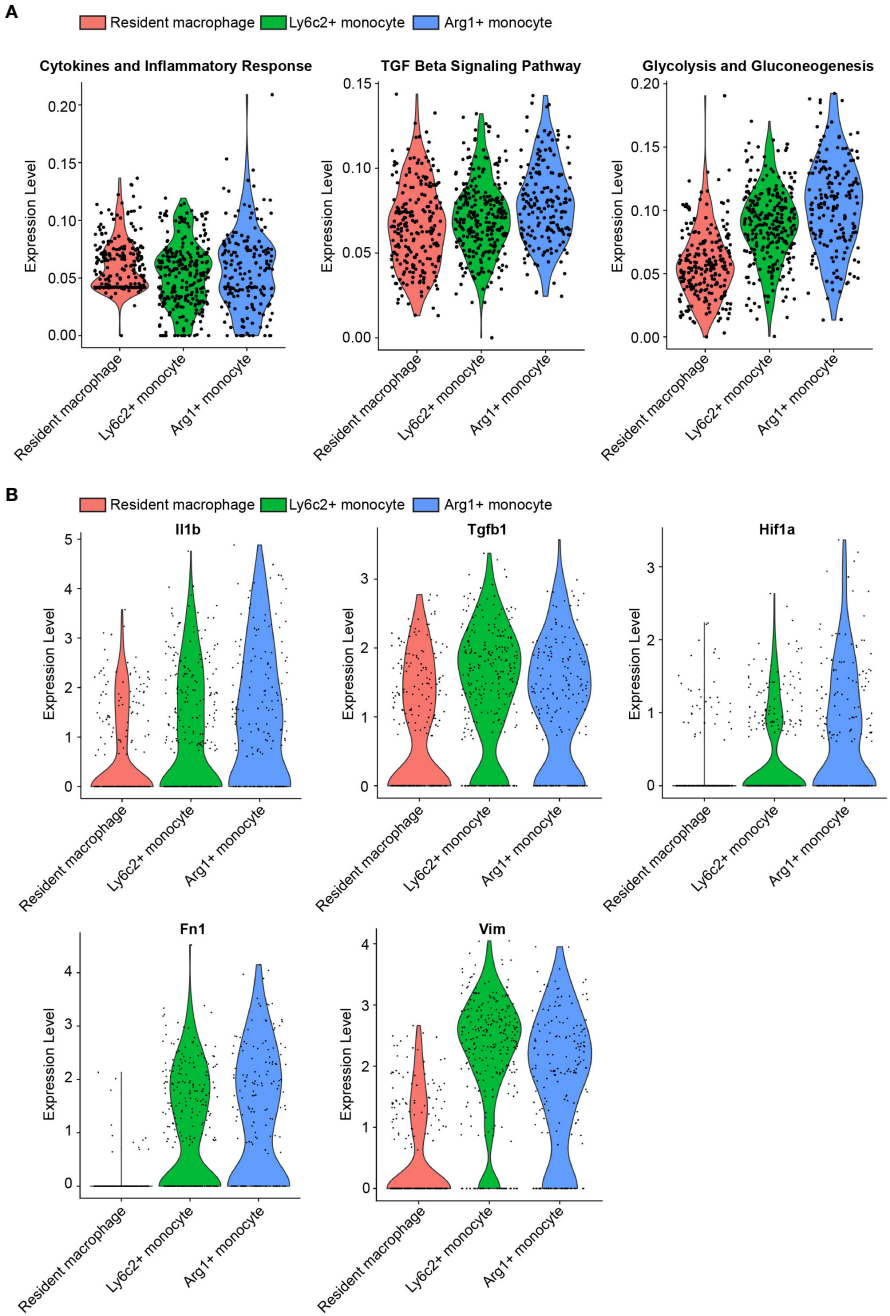
scRNAseq reveals upregulated glycolysis in myeloid cells infiltrating in the UUO kidney. **(A)** Violin plots of inflammation, TGFβ signaling, and glycolysis pathways in resident macrophage, Ly6c2+ macrophage and Arg1+ monocyte clusters. **(B)** Violin plots of indicated genes in resident macrophage, Ly6c2+ macrophage and Arg1+ monocyte clusters.

In a study by Conway et al., myeloid populations crucial for renal fibrosis in the UUO kidney were characterized using single-cell RNA sequencing (scRNAseq) (50). Among these myeloid populations, the significance of Ly6c2^+^ and Arg1^+^ myeloid cells in renal fibrosis were observed (50). To gain further insights of the early events in kidney obstruction, we conducted a reanalysis of the inflammation, TGFβ signaling, and glycolysis related mRNA expression in these myeloid cells and compared them to resident macrophages in UUO 2-day condition. Our findings revealed substantially higher levels of mRNA expression in inflammation, TGFβ signaling, and glycolysis pathways in Ly6c^+^ and Arg1^+^ myeloid cells than those of resident macrophages (**Figure 1A**, **Supplementary Figure 1**). Consistent with these altered pathways, the corresponding genes involved in these pathways were upregulated in Ly6C^+^ and Arg1^+^ myeloid cells compared to resident macrophages. Notably, these genes included Il1, Tgfb1, and Hif1 (**Figure 1B**, **Supplementary Figure 2**). Intriguingly, Ly6C^+^ and Arg1^+^ myeloid cells also exhibited elevated levels of Fn1 and Vim in comparison to resident macrophages (**Figure 1B**), suggesting a potential role of myeloid glycolysis in renal fibrosis initiation.

### Decreased renal fibrosis in myeloid-specific *Pfkfb3*-deficient mice following UUO

**Figure 2:**
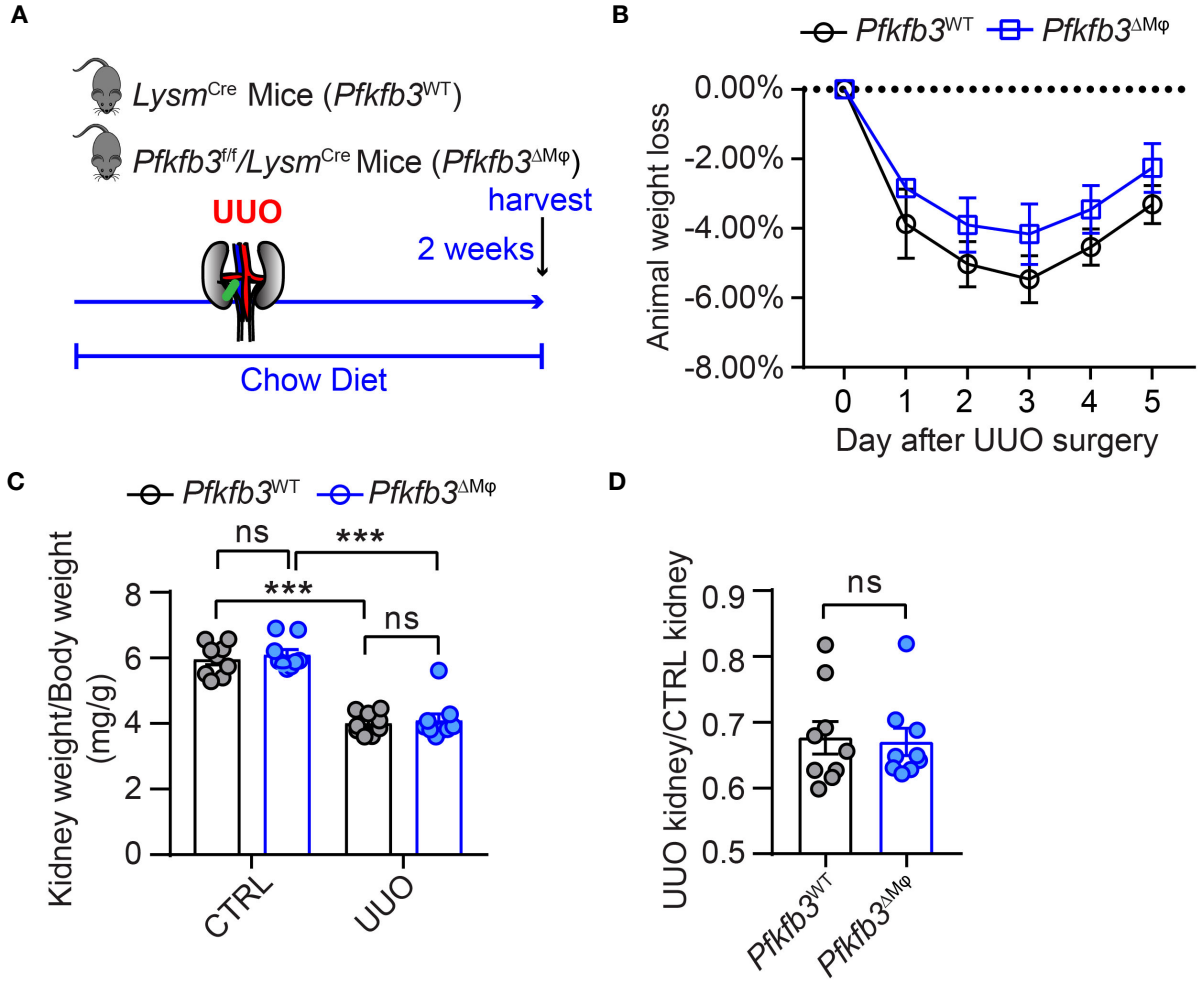
The myeloid *Pfkfb3* deficiency does not show significant impact on body weight and kidney weight loss after ureter obstruction. **(A)** Schematic illustration of Unilateral ureteral obstruction (UUO) in myeloid *Pfkfb3* deficient mice. **(B)** The daily body weight loss percentage for *Pfkfb3* ^ΔMφ^ and *Pfkfb3* ^WT^ mice subjected to UUO surgery for 5 days. **(C)** The ratio of kidney weight to body weight from *Pfkfb3* ^ΔMφ^ and *Pfkfb3* ^WT^ mice subjected to UUO surgery for 14 days. **(D)** The ratio of fibrotic kidney weight to control from *Pfkfb3* ^ΔMφ^ and *Pfkfb3* ^WT^ mice subjected to UUO surgery for 14 days. n = 9 mice per group. Data are means ± SEM. ns, no significance; ***p < 0.001 for indicated comparisons. Statistical significance was determined by one-way ANOVA followed by the Bonferroni test **(C)** or by Student’s *t* test **(D)**.

**Figure 3:**
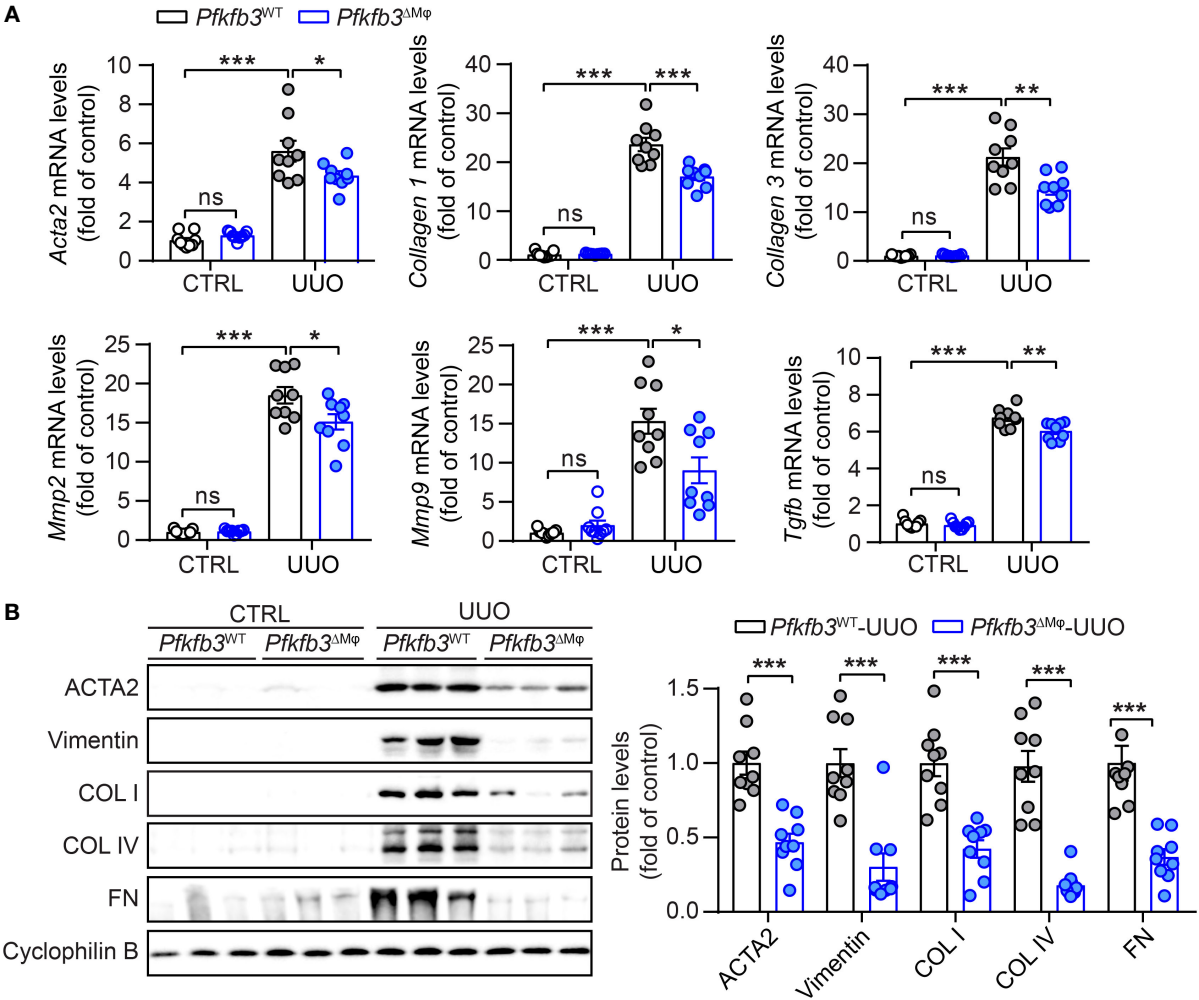
The myeloid *Pfkfb3* deficiency suppresses renal fibrosis in UUO mouse model. **(A)** qRT-PCR analysis of the mRNA expression of *Acta2*, *Col1*, *Col3*, *Mmp2*, *Mmp9* and *Tgfb* in kidney collected from *Pfkfb3* ^ΔMφ^ and *Pfkfb3* ^WT^ mice at day 14 post UUO surgery. **(B)** Representative Western Blots and their quantification showing ACTA2, Vimentin, COL I, COL IV, FN protein expression in kidney collected from *Pfkfb3* ^ΔMφ^ and *Pfkfb3* ^WT^ mice at day 14 post UUO surgery. n = 9 mice per group. Data are means ± SEM. ns, no significance; *p < 0.05; **p < 0.01; ***p < 0.001 for indicated comparisons. Statistical significance was determined by one-way ANOVA followed by the Bonferroni test **(A)** or by Student’s *t* test **(B)**.

To investigate the role of myeloid PFKFB3 in renal fibrosis, we generated myeloid-specific *Pfkfb3*-deficient mice *(Pfkfb3* ^ΔMϕ^ *)* and their controls (*Pfkfb3* ^WT^) by breeding floxed *Pfkfb3* mice with Lysm-Cre mice (**Supplementary Figures 3A, B**). Deletion of *Pfkfb3* in myeloid cells was confirmed through Real time PCR, Western blot analysis and immunostaining of PFKFB3 in Bone marrow derived macrophages (**Supplementary Figures 3C-E**). We conducted unilateral ureter obstruction (UUO) surgery in these mice (**Figure 2A**). The change in mouse body weight following the UUO procedure did not show a significant difference between the two groups (**Figure 2B**). After 14 days, the weight of obstructive kidneys was significantly reduced compared to that of control kidneys, indicating obvious kidney atrophy (**Figures 2C, D**, **Supplementary Figure 4**). And hydronephrosis was observed at kidney harvesting, indicating the success of ureter obstruction. Meanwhile, there was no significant difference of kidney weight between groups of *Pfkfb3* ^ΔMϕ^ and *Pfkfb3* ^WT^ mice (**Figures 2C, D**). The co-staining of PFKFB3 and F4/80 confirmed the induction of PFKFB3 in *Pfkfb3* ^WT^ macrophages and the depletion of *Pfkfb3* in *Pfkfb3* ^ΔMϕ^ macrophages in kidneys after UUO injury (**Supplementary Figure 5**). Using qRT-PCR, we examined the mRNA levels of multiple fibrotic factors. The expression of *Acta2*, *Col*1 and 3, *Mmp*2 and 9, and *Tgfb* were significantly increased in UUO kidneys compared to control kidneys (**Figure 3A**). Notably, the upregulation of these fibrotic factors in UUO kidneys of *Pfkfb3* ^ΔMϕ^ mice was markedly reduced compared to *Pfkfb3* ^WT^ mice (**Figure 3A**). We also assessed the protein levels of a few fibrotic factors through Western blot analysis (**Figure 3B**). The levels of α-smooth muscle actin (ACTA2), vimentin (VIM), collagen (COL) I and IV and fibronectin (FN) were significantly elevated in UUO kidneys compared to control kidneys. However, the increased levels of these fibrotic factors in UUO kidneys of *Pfkfb3* ^ΔMϕ^ mice were significantly reduced compared to *Pfkfb3* ^WT^ mice (**Figure 3B**).

**Figure 4:**
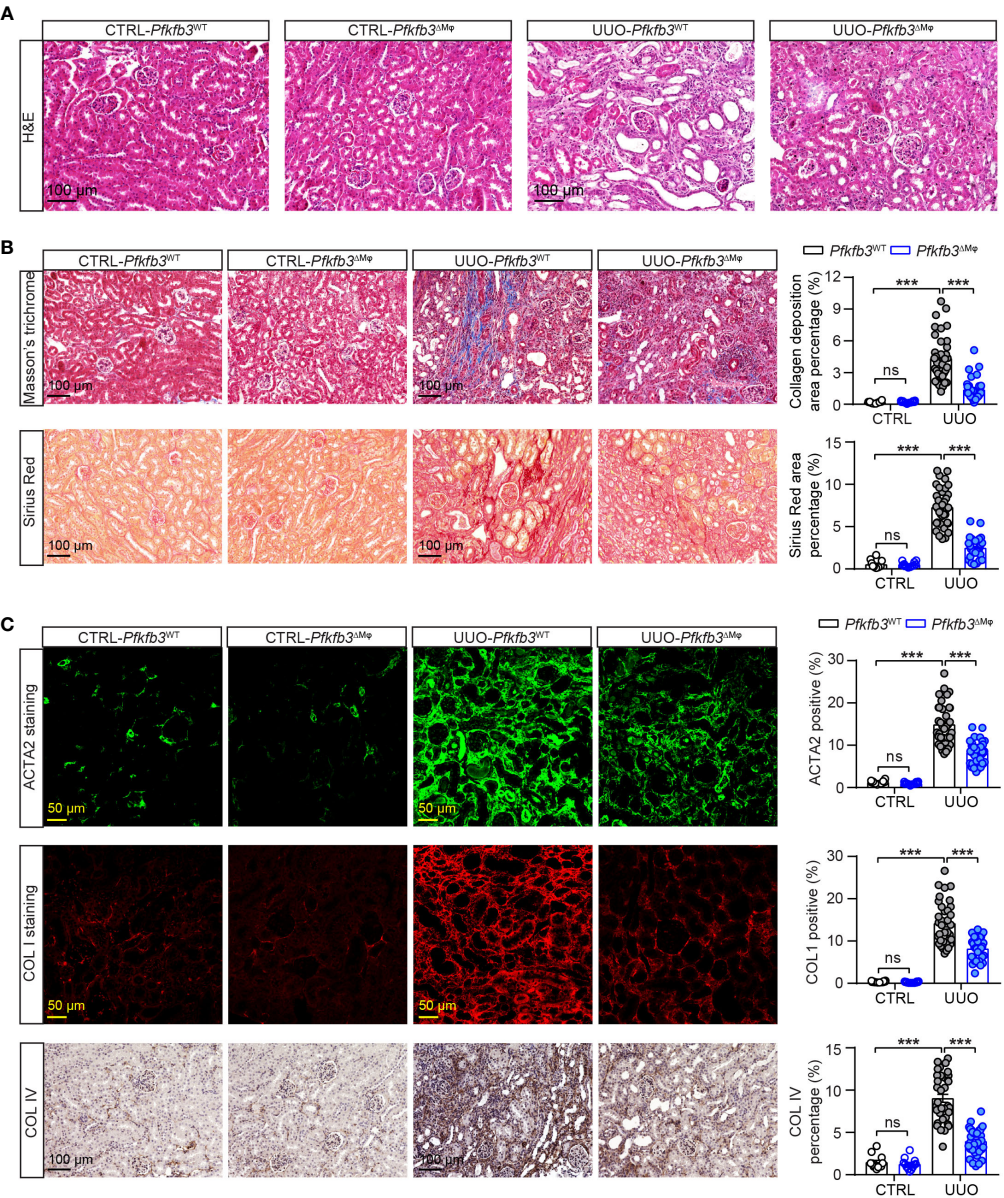
The myeloid *Pfkfb3* deficiency suppresses renal fibrosis in obstructive kidney. *Pfkfb3* ^ΔMφ^ and *Pfkfb3* ^WT^ mice were subject to UUO for 14 days, kidneys were collected and fixed for paraffin-embedded sections. **(A)** Representative image of hematoxylin and eosin (H&E) staining. n = 9 mice/group. **(B)** Representative image and quantification data of Masson’s trichrome staining and Sirius red staining. **(C)** Representative image and quantification data of ACTA2, COL1 and COL IV staining. Scale bar = 100 μm or 50 μm. n = 3 mice/control group and n = 9 mice/UUO group, 4 areas/section were quantified. Data are means ± SEM. ns, no significance; ***p < 0.001 for indicated comparisons. Statistical significance was determined by one-way ANOVA followed by the Bonferroni test.

Furthermore, we verified the interstitial renal fibrosis by staining renal sections of UUO and control kidneys with Sirius Red and Masson’s trichrome. In the UUO kidneys from *Pfkfb3* ^WT^ mice, characterized by pronounced tubular dilation and thinning on hematoxylin and eosin (HE) stained sections, there was evident collagen deposition with Masson’s trichrome and Sirius Red staining, indicating a significant interstitial renal fibrosis (**Figures 4A, B**, **Supplementary Figure 4**). In contrast, there were fewer tubular destruction and less collagen deposition shown by Masson’s trichrome and Sirius Red staining in the sections of UUO kidneys from *Pfkfb3* ^ΔMϕ^ mice compared to *Pfkfb3* ^WT^ mice (**Figures 4A, B**).

We also performed immunostaining to examine the presence of specific myofibroblast marker and components of extracellular matrix. As shown in **Figure 4C**, the levels of ACTA2, Collagen I and IV, which were low but detectable in the control kidneys from both groups of mice, were remarkably elevated in the UUO kidneys of *Pfkfb3* ^WT^ mice. However, these increased levels of myofibroblast marker and extracellular matrix proteins were significantly reduced in the UUO kidneys from *Pfkfb3* ^ΔMϕ^ mice (**Figure 4C**).

### Declined number of renal macrophages in myeloid-specific *Pfkfb3*-deficient mice following UUO

**Figure 5:**
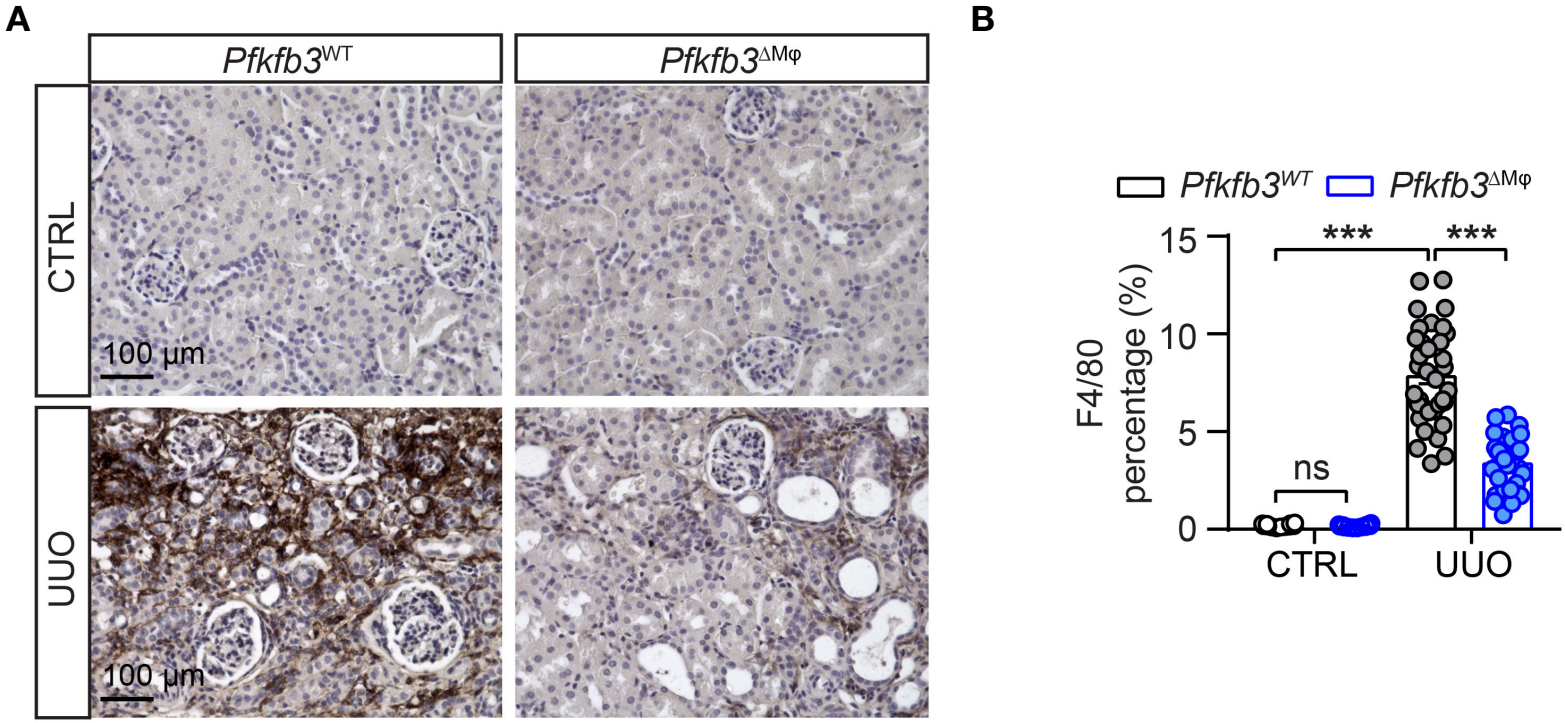
Declined number of macrophages in myeloid-specific *Pfkfb3*-deficient mice following UUO. *Pfkfb3* ^ΔMφ^ and *Pfkfb3* ^WT^ mice were subject to UUO for 14 days, kidneys were collected and fixed for paraffin-embedded sections. **(A)** Representative image of F4/80 staining. Scale bar = 100 μm. **(B)** Quantification data of F4/80 staining. n = 3 mice/control group and n = 9 mice/UUO group, 4 areas/section/kidney were quantified. The percentage of F4/80 staining was determined by calculating the ratio of the F4/80-positive area to the total area, observed under a 20X microscope objective. Data are means ± SEM. ns, no significance; ***p < 0.001 for indicated comparisons. Statistical significance was determined by one-way ANOVA followed by the Bonferroni test.

We investigated the infiltration of macrophages in the UUO kidney of *Pfkfb3* ^ΔMϕ^ mice by performing immunostaining on renal sections using the F4/80 antibody. The presence of F4/80 positive cells was not noticeable in control kidneys from both *Pfkfb3* ^WT^ and *Pfkfb3* ^ΔMϕ^ mice (**Figures 5A, B**). However, an obvious increase of F4/80-positive cells was observed in the UUO *Pfkfb3* ^WT^ kidneys (**Figures 5A, B**). Remarkably, the F4/80 positive area was significantly reduced in the UUO *Pfkfb3* ^ΔMϕ^ kidney sections compared to the UUO *Pfkfb3* ^WT^ kidney (**Figures 5A, B**). These findings strongly suggest that myeloid *Pfkfb3* deficiency markedly inhibits the infiltration of macrophages into the UUO kidneys.

### Reduced markers of M1 and M2 macrophages and related cytokines in myeloid-specific *Pfkfb3*-deficient mice following UUO

**Figure 6:**
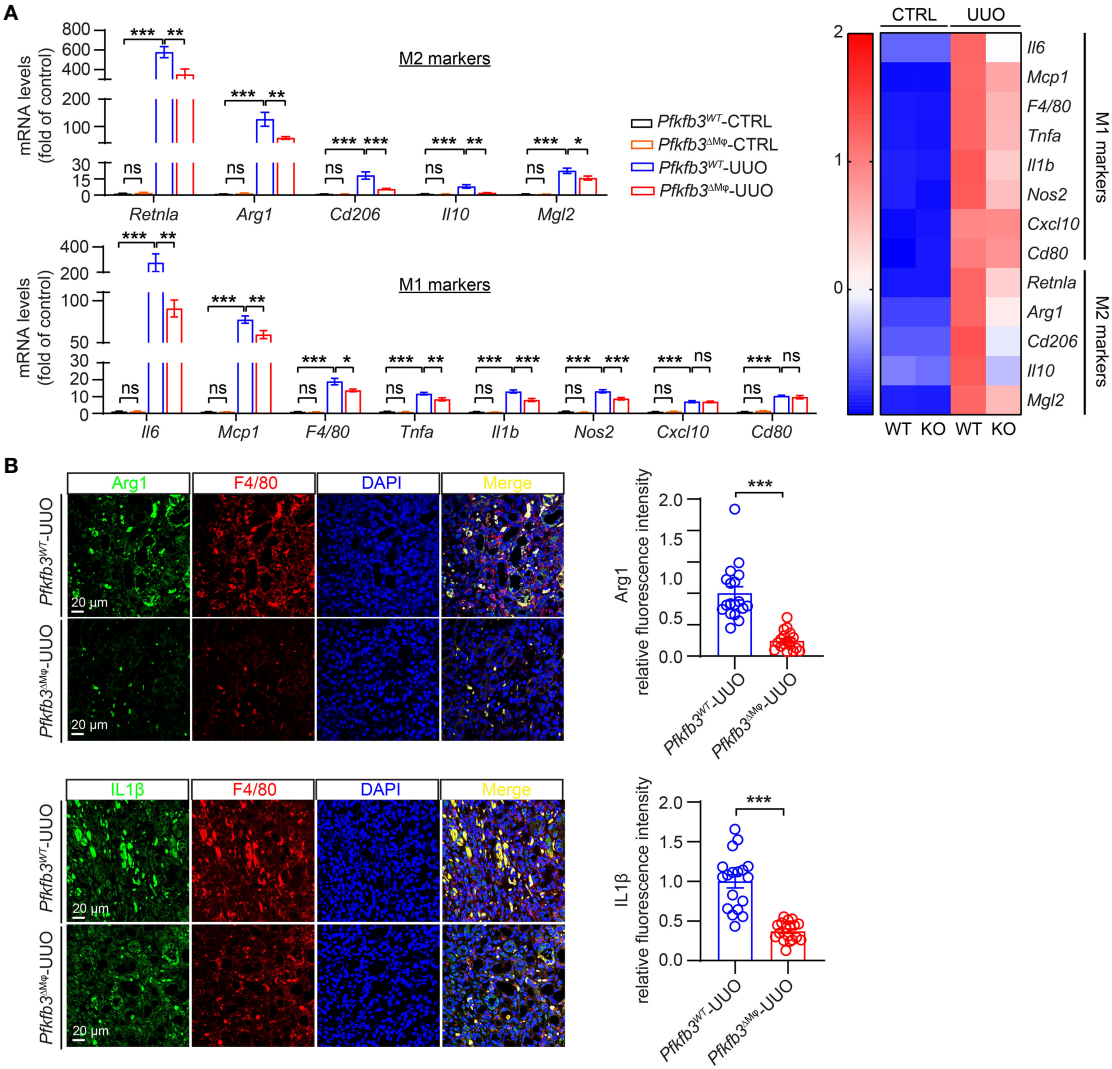
Reduced markers of M1 and M2 macrophages and related cytokines in myeloid-specific *Pfkfb3*-deficient mice following UUO. **(A)** qRT-PCR analysis of the mRNA expression of indicated genes in kidney collected from *Pfkfb3* ^ΔMφ^ and *Pfkfb3* ^WT^ mice at day 14 post UUO. n = 9 mice/group. In the heatmap, Z-scores were calculated for each gene. **(B)** Representative images and the relative intensity quantification of Arg1 and IL1β staining in kidney collected from *Pfkfb3* ^ΔMφ^ and *Pfkfb3* ^WT^ mice at day 14 post UUO surgery. Nuclei were counterstained with DAPI (blue). n = 9 mice/group. Scale bar = 100 μm. Data are means ± SEM. ns, no significance; *p < 0.05; **p < 0.01; ***p < 0.001 for indicated comparisons. Statistical significance was determined by one-way ANOVA followed by the Bonferroni test.

To assess the impact of myeloid *Pfkfb3* deficiency on the infiltration of M1 and M2 macrophages in the UUO kidneys, we examined the mRNA and protein levels of M1/M2 markers and cytokines associated with M1/M2 macrophages. As shown in **Figure 6A**, the expression levels of M2 markers *Arg1*, *Cd206*, and M1 markers *Cd80* were detectable but similar in the control kidneys of both groups of mice. However, in the UUO *Pfkfb3* ^WT^ kidney, there was a substantial upregulation of both M2 and M1 markers (**Figure 6A**). In contrast, these markers were significantly reduced in the UUO *Pfkfb3* ^ΔMϕ^ kidney compared to the UUO *Pfkfb3* ^WT^ kidney (**Figure 6A**). Consistent with the levels of M1/M2 markers, the levels of cytokines, including Il10, Mgl2, Retnla, Il6, Mcp1, Tnfa, Il1b, Nos2, and Cxcl10, were significantly lower in the UUO *Pfkfb3* ^ΔMϕ^ kidney compared to the UUO *Pfkfb3* ^WT^ kidney (**Figure 6A**). To validate the expression of some genes at protein levels, we examined the expression of Arg1 and Il1β on F4/80 positive cells by immunostaining in renal sections with their specific antibodies. The staining intensity of Arg1 and Il1β on F4/80-positive cells were much lower in sections of the UUO *Pfkfb3* ^ΔMϕ^ kidney compared to the UUO *Pfkfb3* ^WT^ kidney (**Figure 6B**). These findings indicate that *Pfkfb3* deficiency leads to a significant reduction in the number of M1 and M2 macrophages, as well as the levels of cytokines associated with these macrophage phenotypes in UUO kidneys.

### Lowered macrophage differentiation in myeloid-specific *Pfkfb3*-deficient obstructive kidneys

**Figure 7:**
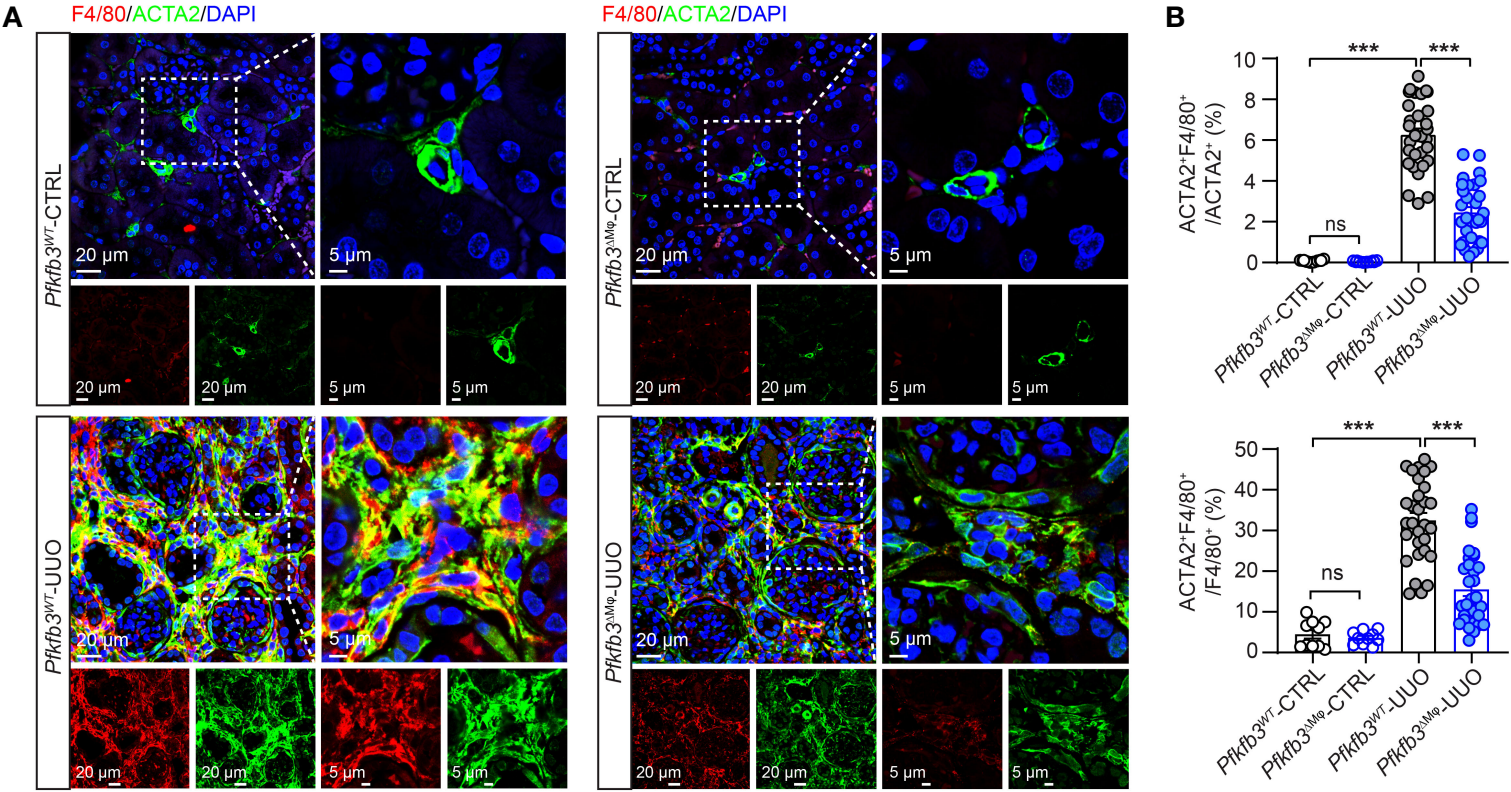
Reduced macrophage to myofibroblast cell transition (MMT) in myeloid-specific *Pfkfb3*-deficient mice following UUO. *Pfkfb3* ^ΔMφ^ and *Pfkfb3* ^WT^ mice were subject to UUO for 14 days, kidneys were collected and fixed for paraffin-embedded sections. **(A)** Representative image of ACTA2 and F4/80 staining. Nuclei were counterstained with DAPI (blue). Scale bar = 20 μm and 5 μm. **(B)** Quantification of the percentages of ACTA2 and F4/80 co-stained cells in myofibroblasts (ACTA2+) or macrophage (F4/80+). n = 3 mice/sham group and n = 9 mice/UUO group, 4 areas/section/kidney were quantified. Data are means ± SEM. ns, no significance; ***p < 0.001 for indicated comparisons. Statistical significance was determined by one-way ANOVA followed by the Bonferroni test.

To investigate whether myeloid *Pfkfb3* deficiency affected macrophage differentiation to obtain myofibroblast phenotype in the UUO kidney, we quantified the number of macrophages expressing ACTA2 through co-immunostaining of the renal sections. In the control kidneys, the presence of ACTA2 and F4/80 positive cells was rare (**Figures 7A, B**). Conversely, the UUO *Pfkfb3* ^WT^ kidneys exhibited a significant induction of F4/80 positive cells, with about 6% of them co-stained with ACTA2 (**Figures 7A, B**), suggesting their transition to myofibroblasts. Remarkably, the UUO *Pfkfb3* ^ΔMϕ^ kidneys showed a substantial reduction of F4/80 positive area as well as the double-positive area of F4/80 and ACTA2 compared to the UUO *Pfkfb3* ^WT^ kidney (**Figures 7A, B**). Further analysis on the lineage of myofibroblasts revealed that, in the UUO Pfkfb3WT kidneys, about 32% of myofibroblasts (ACTA2 positive) expressed F4/80. This percentage dropped to approximately 15% in the UUO Pfkfb3ΔMϕ kidneys (**Figure 7B**). These findings indicate that myeloid-specific *Pfkfb3* deficiency leads to a decrease of differentiated macrophage with myofibroblast characteristics in the UUO kidney.

### Decreased TGFβ1-induced M1 and M2 markers, cytokines and macrophage differentiation with *Pfkfb3* deficiency

**Figure 8:**
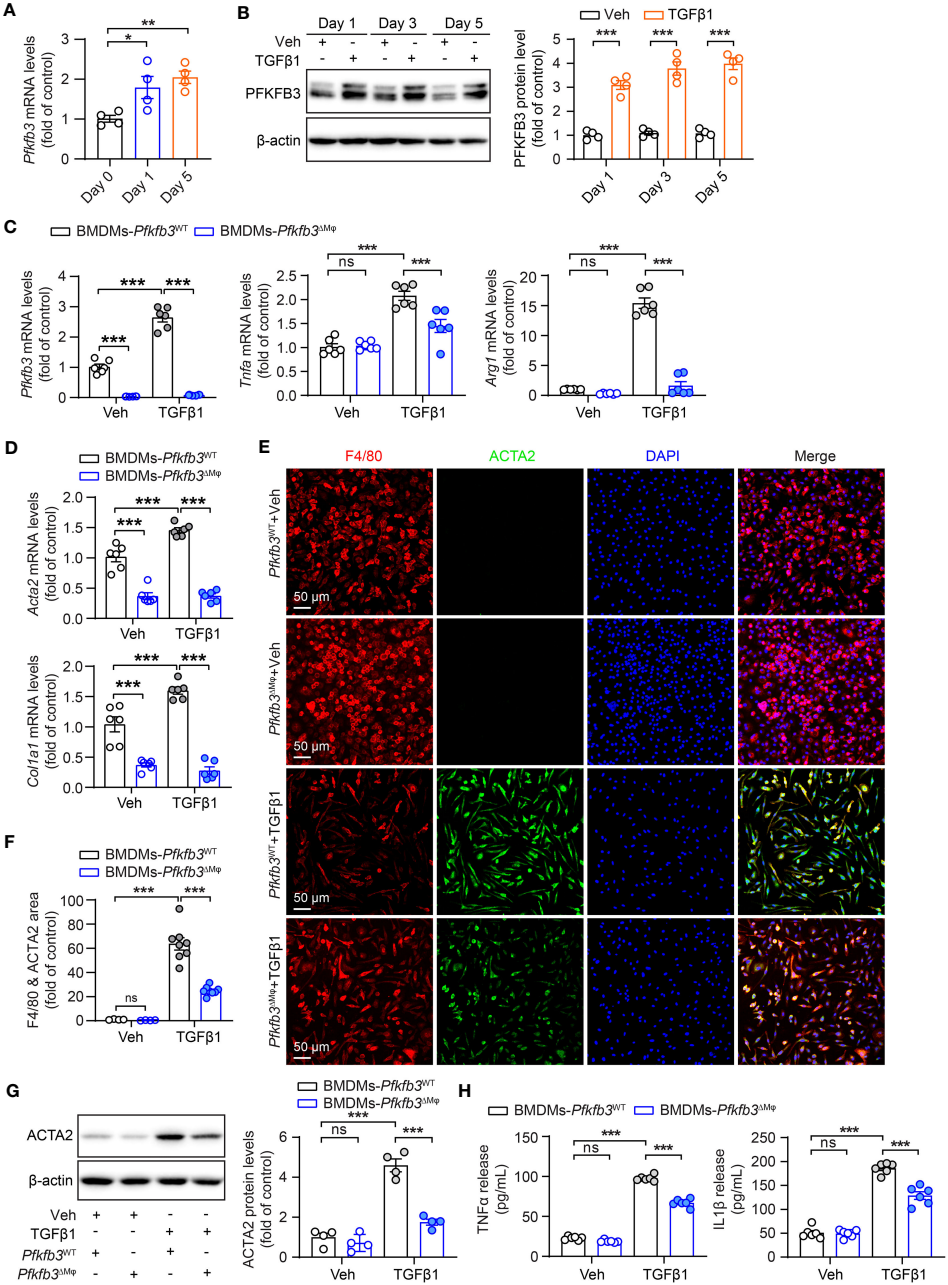
Decreased TGFβ1-induced cytokine production and MMT of *Pfkfb3* deficient macrophages. **(A)** qRT-PCR analysis of the mRNA expression of *Pfkfb3* in BMDMs cultured from *Pfkfb3* ^WT^ mice and treated with TGFβ1 or vehicle for 1 day and 5 days. n = 4. **(B)** Representative Western Blots and their quantification showing PFKFB3 protein levels in BMDMs cultured from *Pfkfb3* ^WT^ mice and treated with TGFβ1 or vehicle for 1 day, 3 days and 5 days. n = 4. **(C)** qRT-PCR analysis of the mRNA expression of *Pfkfb3*, *Tnfa* and *Arg1* in BMDMs cultured from *Pfkfb3* ^WT^ or *Pfkfb3* ^ΔMφ^ mice and treated with TGFβ1 for 5 days. n = 6. **(D)** qRT-PCR analysis of the mRNA expression of *Acta2* and *Col1* in BMDMs cultured from *Pfkfb3* ^WT^ or *Pfkfb3* ^ΔMφ^ mice and treated with TGFβ1 for 5 days. n = 6. **(E, F)** Representative image and quantification data of ACTA2 and F4/80 staining area percentage of BMDMs cultured from *Pfkfb3* ^WT^ or *Pfkfb3* ^ΔMφ^ mice and treated with TGFβ1 for 5 days. **(G)** Representative immunoblots and densitometry quantification of ACTA2 in BMDMs cultured from *Pfkfb3* ^WT^ or *Pfkfb3* ^ΔMφ^ mice and treated with TGFβ1 for 5 days. n = 4. **(H)** Measurement of TNFα and IL1β in BMDMs culture medium by ELISA. n = 6. Data are means ± SEM. ns, no significance; *p < 0.05; **p < 0.01; ***p < 0.001 for indicated comparisons. Statistical significance was determined by one-way ANOVA followed by the Bonferroni test **(A, C, D, F-H)** or by Student’s *t* test **(B)**.

After observing the phenotypic changes of M1 and M2 markers, cytokines, and myofibroblast maker in the UUO *Pfkfb3* ^ΔMϕ^ kidney, we further examined whether *Pfkfb3* deficiency could induce similar changes in macrophages *in vitro.* We first isolated bone marrow cells from *Pfkfb3* ^WT^ and *Pfkfb3* ^ΔMϕ^ mice and cultured bone marrow-derived macrophages (BMDMs). Upon TGFβ1 treatment, the expression of Pfkfb3 at both mRNA and protein levels was enhanced in BMDMs from *Pfkfb3* ^WT^ mice (**Figures 8A, B**). Subsequently, TGFβ1 incubation significantly upregulated the mRNA levels of M1 and M2 markers and associated cytokines, such as *Tnfa* and *Arg1* (**Figure 8C**). In contrast, *Pfkfb3* deficient BMDMs exhibited significantly lower levels of these mRNAs compared to WT BMDMs with TGFβ1 (**Figure 8C**). Furthermore, TGFβ1 treatment resulted in the differentiation of BMDMs, as evidenced by the expression of Acta2 at both mRNA and protein levels in the TGFβ1-treated group but not in the vehicle-treated group, and it was associated with *Col1a1* mRNA change (**Figures 8D-G**). Remarkably, TGFβ1-induced Acta2 expression was nearly abolished in *Pfkfb3* deficient BMDMs (**Figures 8D-G**). We subsequently analyzed the release of pro-inflammatory cytokines, TNFα and IL1β, by the BMDMs into the culture medium. As depicted in **Figure 8H**, TGFβ1 triggered a substantial release of these cytokines, which was markedly attenuated by the PFKFB3 knockout.

### Involvement of HIF1α in PFKFB3-regulated macrophage phenotypic change

**Figure 9:**
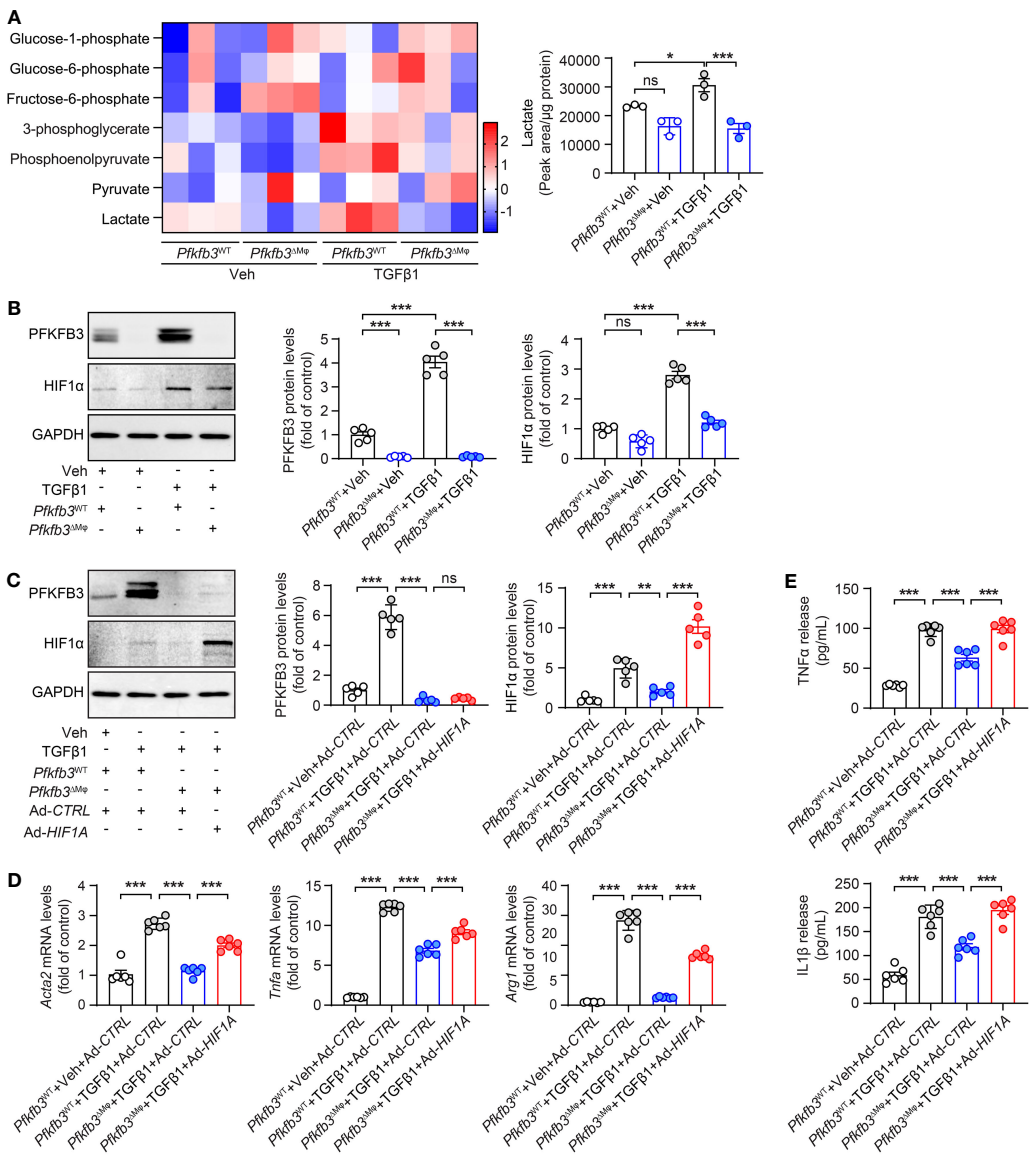
Pfkfb3 regulated the expression of pro-inflammatory cytokines through Hif1a. **(A)** Heat map showing the metabolites in the glycolysis pathway and quantification data of lactate level in BMDMs cultured from *Pfkfb3* ^WT^ or *Pfkfb3* ^ΔMφ^ mice and treated with TGFβ1 for 24 h. n = 3. **(B)** Representative Western Blots and their quantification showing PFKFB3 and HIF1α protein levels in BMDMs cultured from *Pfkfb3* ^WT^ or *Pfkfb3* ^ΔMφ^ mice and treated with TGFβ1 for 5 days. n = 5. **(C)** Representative Western Blots and their quantification showing PFKFB3 and HIF1α protein levels in BMDMs cultured from *Pfkfb3* ^WT^ or *Pfkfb3* ^ΔMφ^ mice transfected with Ad-*CTRL* or Ad- *HIF1α* adenovirus and treated with TGFβ1 for 5 days. n = 5. **(D)** qRT-PCR analysis of the mRNA expression of *Acta2*, *Tnfa* and *Arg1* in BMDMs cultured from *Pfkfb3* ^WT^ or *Pfkfb3* ^ΔMφ^ mice transfected with Ad-*CTRL* or Ad- *HIF1α* adenovirus and treated with TGFβ1 for 5 days. n = 6. **(E)** Measurement of TNFα and IL1β in culture medium by ELISA. n = 6. Data are means ± SEM. ns, no significance; *p < 0.05; **p < 0.01; ***p < 0.001 for indicated comparisons. Statistical significance was determined by one-way ANOVA followed by the Bonferroni test.

We assessed glucose metabolism in cultured WT and *Pfkfb3*-deficient BMDMs using liquid chromatography-tandem mass spectrometry (LC-MS/MS). *Pfkfb3*-deficient BMDMs displayed a modest decrease in the levels of some glycolytic metabolites compared with vehicle-treated WT BMDMs (**Figure 9A**). Upon TGFβ1 treatment, WT BMDMs exhibited a substantial increase in glycolytic metabolites, whereas *Pfkfb3*-deficient BMDMs failed to show similar increases (**Figure 9A**). Most importantly, the increase of the glycolysis end-product lactate relied on PFKFB3 induction (**Figure 9A**). Additionally, we observed the PFKFB3-dependent glycolysis level change after TGFβ1 by Seahorse analysis of ECAR (data not shown).

Since glycolytic metabolites are known to stabilize HIFs and modulate the phenotypic change of vascular cells (51), we investigated the protein level of HIF1α in our cultured BMDMs. The level of HIF1α was comparable between vehicle treated WT and *Pfkfb3*-deficient BMDMs (**Figure 9B**). However, TGFβ1 treatment significantly elevated the level of HIF1α in WT BMDMs (**Figure 9B**), while this upregulation was compromised in *Pfkfb3*-deficient BMDMs (**Figure 9B**). To determine whether this decreased HIF1α in TGFβ1-treated *Pfkfb3*-deficient BMDMs is responsible for the decreased expression of M1 and M2 markers, cytokines and Acta2, we transduced *Pfkfb3*-deficient BMDMs with an adenovirus-packaged non-degradable mutant HIF1α, aiming to restore the HIF1α level in these cells (**Figure 9C**). We measured mRNA levels of M1, M2 macrophage markers, Acta2, and cytokines with qPCR and observed that the decreased expression of these molecules in *Pfkfb3*-deficient BMDMs was enhanced following HIF1α overexpression (**Figure 9D**). Furthermore, it correlated with the cytokine release changes in the culture medium (**Figure 9E**). The ECAR measurement also indicated that HIF1α restoration in *Pfkffb3*-deficent BMDMs partially reversed the glycolysis level (data not shown). These findings indicate that HIF1α, at least partially, participates in PFKFB3-mediated macrophage phenotypic change.

## Discussion

**Figure 10:**
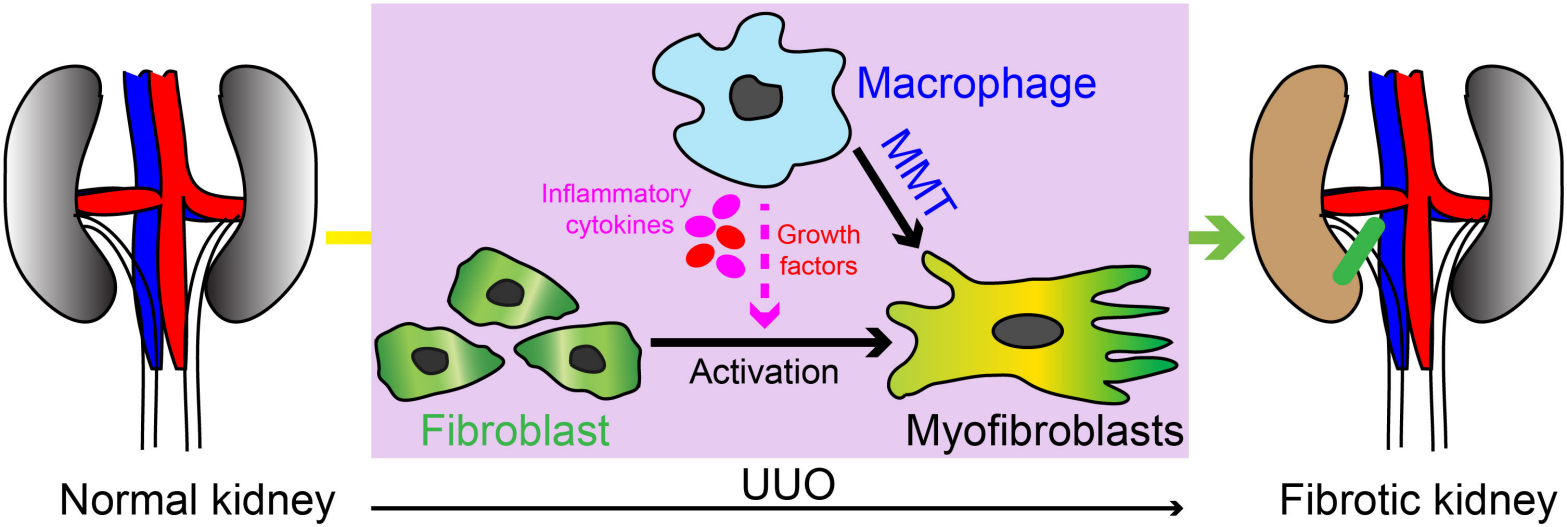
Schematic illustration of the myeloid cell contribution in UUO induced kidney fibrosis. Our study has revealed the crucial involvement of PFKFB3-mediated glycolysis in myeloid cells in the progression of renal fibrosis. The impact of myeloid glycolysis on the development of renal fibrosis encompasses multiple mechanisms, including the modulation of monocyte recruitment, differentiation of M1 and M1 macrophages, and macrophage with myofibroblast phenotype differentiation (also called MMT).

Our study has revealed the crucial involvement of PFKFB3-mediated glycolysis in myeloid cells in the progression of renal fibrosis. The impact of myeloid glycolysis on the development of renal fibrosis encompasses multiple mechanisms, including both the modulation of monocyte recruitment and the differentiation of M1 and M2 macrophages (**Figure 10**).

Monocyte recruitment to the injured kidney is significantly impaired in mice lacking myeloid *Pfkfb3*. The accumulation of monocytes in the kidney is a prominent feature of both acute and chronic kidney disease. This accumulation occurs through the recruitment of circulating monocytes to the kidney, followed by their subsequent proliferation of these recruited monocytes (52, 53). In mice, there are two major subsets of monocytes, named Ly-6C^hi^ and Ly-6C^lo^ (54). Ly-6C^hi^ monocytes are known as inflammatory cells that are actively recruited to the injured kidney (55). The chemokine monocyte chemoattractant protein-1 (MCP-1)/CC-chemokine ligand 2 and its receptor C-C chemokine receptor 2 (CCR2) plays a critical role in monocyte recruitment. Blocking this pathway, either through the deletion of Mcp-1 or using a CCR2 antagonist, suppresses renal injury and the associated renal fibrosis (25, 56, 57). Once infiltrated into the kidney, monocytes differentiate to M1 macrophages and proliferate locally via MCSF/cfms pathway. Inhibiting macrophage proliferation using antibodies against MCSF and c-fms also hinders renal injury and kidney fibrosis (58–62). PFKFB3 is predominantly expressed in Ly-6C^hi^ myeloid cells and provides energy for cell motility (38). Loss of *Pfkfb3* impaired myeloid cell infiltration into the vessel wall and lung (37, 38). Using a similar mechanism, myeloid cells may also have a compromised ability to recruit to the injury kidney. Furthermore, PFKFB3-mediated glycolysis supplies metabolic intermediates for biomass generation, which is essential for myeloid proliferation (38). In the absence of *Pfkfb3*, local infiltrated macrophages may experience impaired proliferation. This may explain why the number of macrophages in the UUO kidney was dramatically reduced (**Figure 5**).

Deficiency of *Pfkfb3* suppresses both M1 and M2 macrophages in the UUO kidney. Both M1 and M2 macrophages play significant roles in the development of renal fibrosis (12). M1 macrophages are typically present in early stage of kidney injury (27, 63, 64), and their detrimental impact on kidney injury and fibrosis have been demonstrated in studies where accelerated renal injury occurred upon infusion of M1 macrophages in mice (65), whereas reduced renal injury was observed in mice with depletion of M1 macrophages (66, 67). M1 macrophages exacerbate renal inflammation through the release of proinflammatory cytokines (12, 68) and contribute to renal injury through the release of matrix metalloproteinases (MMPs) (69, 70). In addition to M1 macrophages, M2 macrophages also plays a crucial role in renal fibrosis (12). M2 macrophages expressing CD206 and/or CD163 have been closely associated with kidney fibrosis in human kidney diseases (71, 72) as well as animal kidney diseases (73–76). Depletion of M2 macrophages in experimental setups has been shown to protect rodents from fibrosis, while adoptive transfusion of M2 macrophages to rodents with renal injury has accelerated kidney fibrosis (29, 77), thus demonstrating the causal role of M2 macrophages in the development of kidney fibrosis. In our study, both *in vivo* renal samples and *in vitro* experiments using cultured BMDMs consistently showed decreased levels of both M1 and M2 macrophages, indicating a dual role of PFKFB3-mediated glycolysis in regulating M1 and M2 macrophage phenotypes. The role of PFKFB3-mediated glycolysis in myeloid cells in renal fibrosis aligns with its role in myeloid cells in pathological angiogenesis (36). In hypoxic angiogenesis, *Pfkfb3* deficiency in myeloid cells reduces both M1 and M2 markers, as well as related cytokines/growth factors (36).

The induction of macrophage phenotype differentiation in the kidneys of mice with UUO is significantly hindered in mice lacking myeloid *Pfkfb3*. A notable portion of fibroblasts in fibrotic kidneys in both humans and animals exhibit markers associated with bone marrow cells and/or CD68 (4, 22, 78). This population of cells may arise from a further differentiation of M2, as evidenced by the expression of M2 markers including CD206 (22). Over the past few years, several studies conducted by different research groups have reported the presence of MMT and its critical role in kidney fibrosis (14, 22, 23, 79, 80). However, recent single-cell sequencing data does not provide substantial evidence supporting a significant contribution of myofibroblasts originating from macrophages (10). And the myofibroblast population is characterized by their production of extracellular matrix (10). It is important to note that this macrophage phenotypic change is also accompanied by a notable alteration in cytokine production (**Figure 8**), suggesting an important regulatory function of these cells in renal fibrosis, despite their direct involvement in extracellular matrix production.

Notably, macrophage is not the only source of myofibroblasts in UUO kidneys. Other cells such as resident renal fibroblast and pericytes also contribute to the myofibroblasts activation (7, 9). Concurrently, decreased macrophage infiltration leads to less pro-inflammatory cytokines release which can regulate all renal myofibroblast activation. Overall, macrophages influence renal fibrosis through both their differentiation into myofibroblasts and their role in inflammatory regulation (19). In our study, we observed the decrease of macrophage-originated myofibroblasts and reduced pro-inflammatory cytokines release in PFKFB3 deficient kidney after UUO injury. However, we do not have conclusive evidence indicating which of these mechanisms is predominant.

Our study also implicates that PFKFB3 may act as a bridge to mediate the crosstalk of TGF-β1 and HIF-1α signaling pathways in renal fibrosis (**Figures 8**, **9**). The regulatory role of PFKFB3-mediated glycolysis in fibrotic activity of TGFβ has been demonstrated in pulmonary fibroblast (81). In line with previous study with fibroblasts (81), the TGFβ-induced expression of αSMA and extracellular matrix, such as collagen, is significantly suppressed in myeloid cells both in mouse kidney and *in vitro* cultured BMDMs in the absence of *Pfkfb3*(**Figure 8**). This suggests a similarity in the regulatory role of PFKFB3 in TGFβ-mediated pathways in different cell types. In myeloid cells, several pathways have been identified as being regulated by glycolytic metabolites (33). For instance, the deficiency of *Pfkfb3* in macrophages resulted in decreased levels of Acetyl-CoA, leading to reduced histone acetylation and subsequently suppressing the transcription of growth factors and proinflammatory cytokines (36). Additionally, a decline in the protein level of phosphor-NF-kB p65 was observed in *Pfkfb3*-deficient BMDMs (35). Glycolytic metabolites have also been shown to stabilize HIFs in vascular cells and macrophages (82, 83). Our study indicates that HIF1α plays a significant role in the effect of PFKFB3-medaited glycolysis on phenotypic alterations observed in macrophages in renal fibrosis. Under fibrotic conditions, PFKFB3-mediated glycolysis can likewise stabilize HIF1α. The mediatory role of HIF1α in PFKFB3-regulated myeloid phenotypic change was demonstrated through the gain-of-function of HIF1α in *Pfkfb3*-deficient BMDMs (**Figure 9**).

Collectively, this study highlights the critical role of PFKFB3-medicated glycolysis in myeloid cells for the progression of kidney fibrosis (**Figure 10**). The activity of PFKFB3 can be effectively suppressed through knockdown using siRNA or inhibition using small molecules (84). Recent advancements have described the development of tool specifically designed to target macrophages (85). Consequently, the inhibition of myeloid PFKFB3-mediated glycolysis holds great promise as a potential therapeutic strategy for treating kidney fibrosis.

## Data availability statement

The original contributions presented in the study are included in the article/**Supplementary Material**. Further inquiries can be directed to the corresponding authors.

## Ethics statement

The animal study was approved by Institutional Animal Care and Use Committee at Augusta University. The study was conducted in accordance with the local legislation and institutional requirements.

## Author contributions

QY: Conceptualization, Data curation, Formal Analysis, Funding acquisition, Investigation, Methodology, Project administration, Supervision, Validation, Writing – original draft, Writing – review & editing. EH: Conceptualization, Data curation, Formal Analysis, Investigation, Methodology, Writing – review & editing. YC: Data curation, Formal Analysis, Investigation, Methodology, Writing – review & editing. ZZ: Data curation, Formal Analysis, Investigation, Methodology, Writing – review & editing. CD: Data curation, Formal Analysis, Investigation, Methodology, Writing – review & editing. JA: Investigation, Methodology, Supervision, Writing – review & editing. HS: Data curation, Investigation, Methodology, Supervision, Writing – review & editing. QW: Conceptualization, Data curation, Formal Analysis, Funding acquisition, Investigation, Methodology, Project administration, Supervision, Validation, Writing – original draft, Writing – review & editing.
