# Supplementary material for: Myeloid PFKFB3-mediated glycolysis promotes kidney fibrosis
Source: Front Immunol. 2023 Nov 16;14:1259434. doi: 10.3389/fimmu.2023.1259434 (PMC10687406; doi:10.3389/fimmu.2023.1259434)
Supplement: Supplementary file 1 [file DataSheet_1.pdf]

Supporting Figures

Supplementary Figure 1

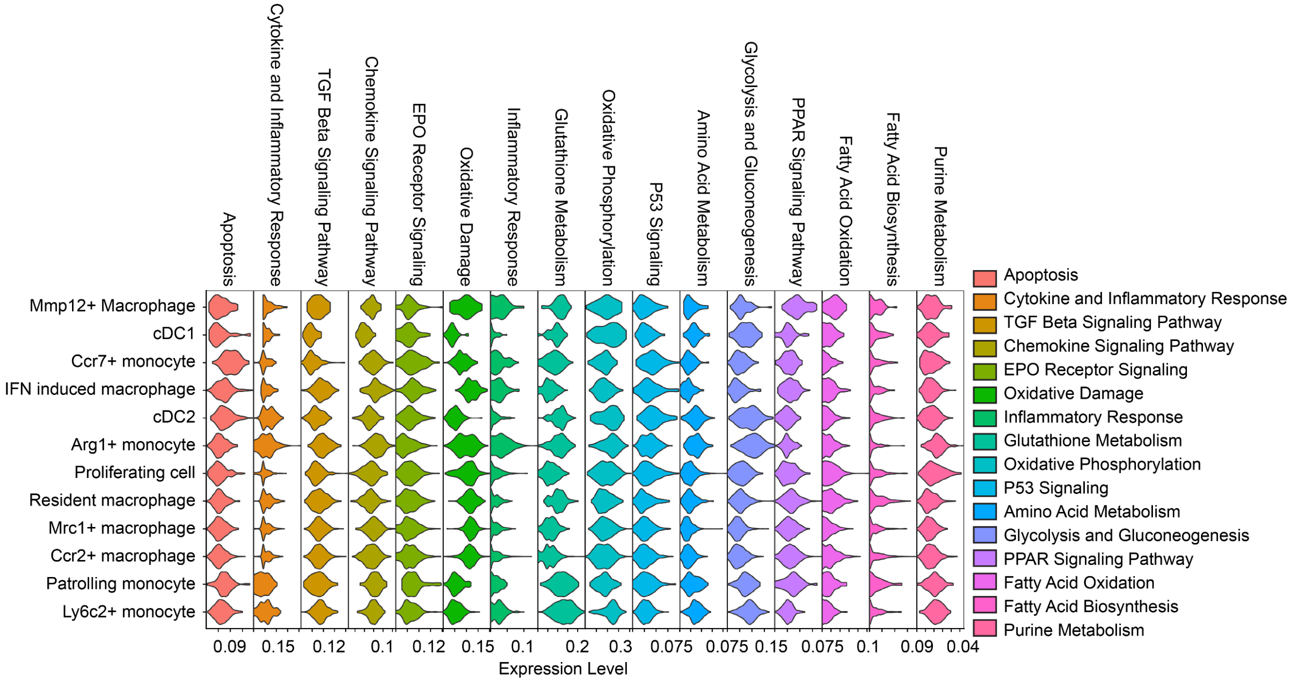

Supplementary Figure 1. Violin plots show the expression levels of selected signaling pathways in each cluster. The x-axis shows the log-scale normalized read count.

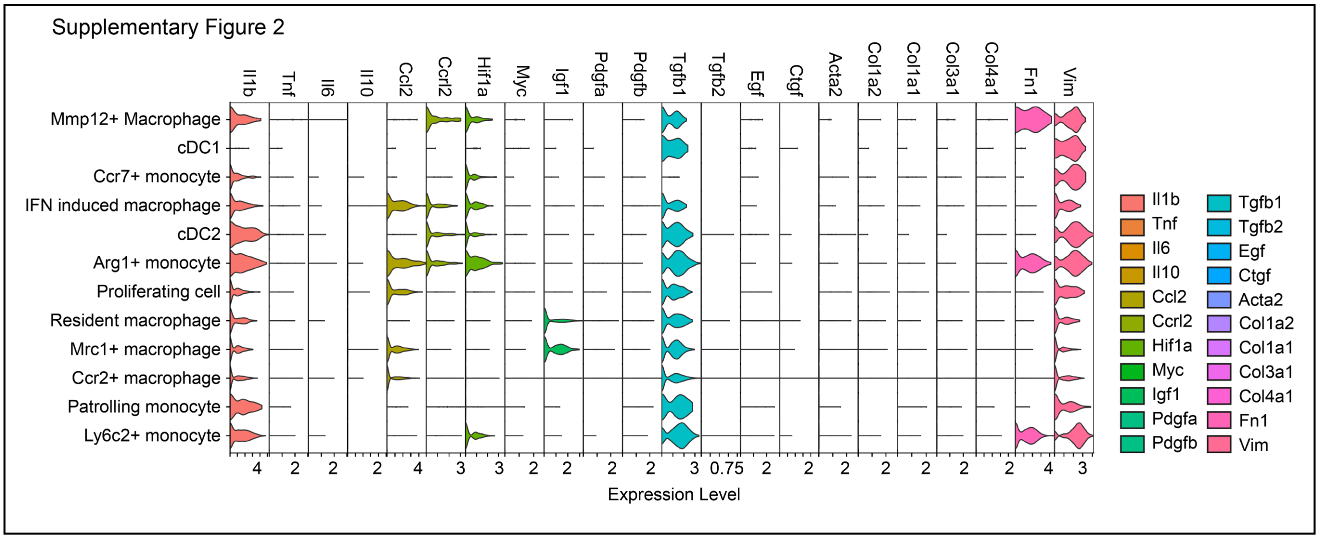

Supplementary Figure 2. Violin plots show the expression levels of selected marker genes in each cluster. The x-axis shows the log-scale normalized read count.

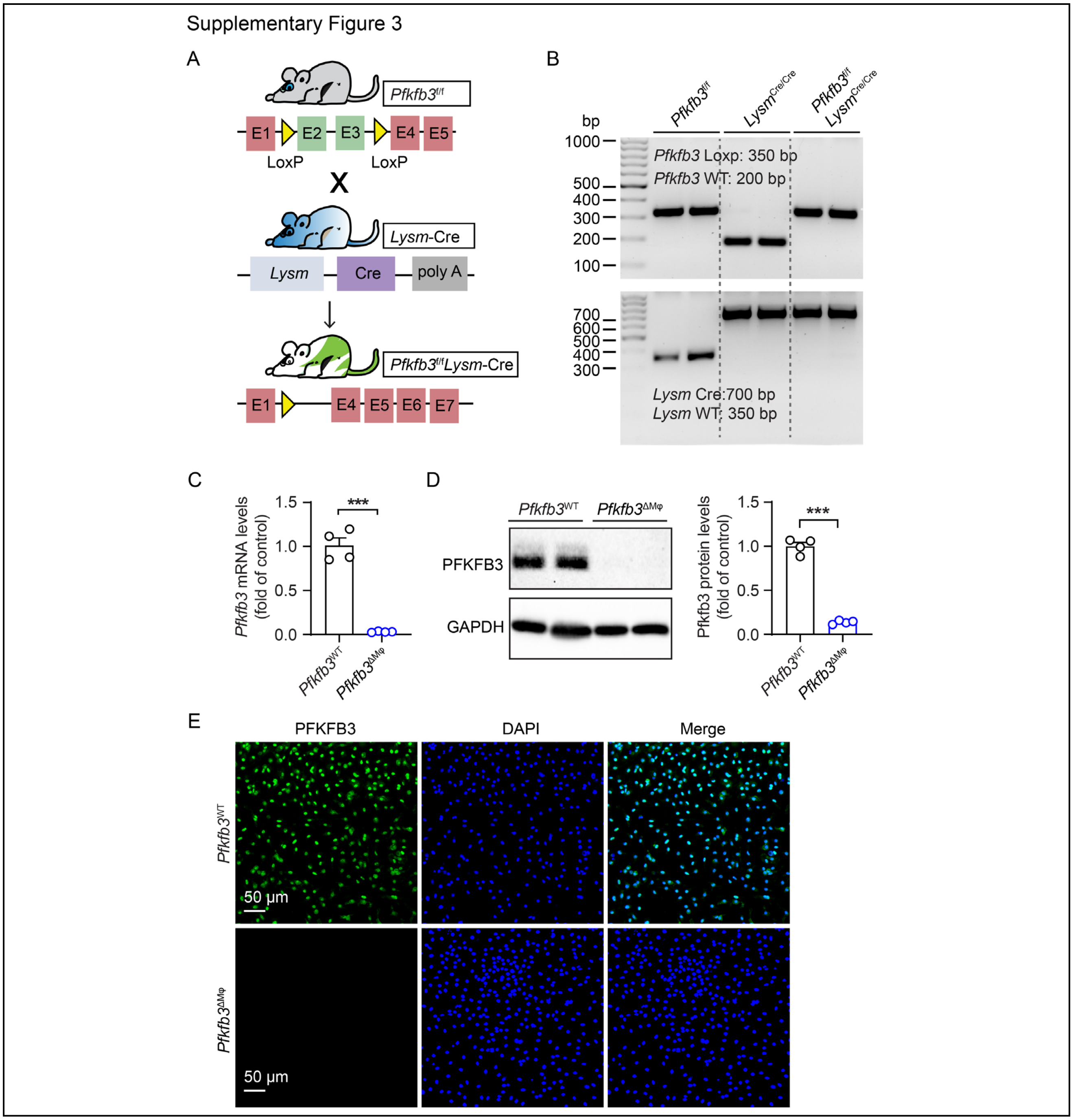

Supplementary Figure 3. Generation and characterization of myeloid-specific *Pfkfb3* deficient mice. **(A)** Schematic illustration of strategy to generate myeloid-specific *Pfkfb3* deficient mice. **(B)** Representative genotyping results for *Pfkfb3*$^{\Delta M\varphi}$ and *Pfkfb3*^{WT} mice. **(C)** qRT-PCR analysis of the mRNA expression of *Pfkfb3* in BMDMs cultured from *Pfkfb3*$^{\Delta M\varphi}$ and *Pfkfb3*^{WT} mice. n = 4. **(D)** Representative Western Blots and the quantification of PFKFB3 protein level in BMDMs cultured from *Pfkfb3*$^{\Delta M\varphi}$ and *Pfkfb3*^{WT} mice. n = 4. **(E)** cultured from *Pfkfb3*$^{\Delta M\varphi}$ and *Pfkfb3*^{WT} mice. Scale bar = 50 µm. Data are means ± SEM. ns, no significance; ***$p < 0.001$ for indicated comparisons. Statistical significance was determined by unpaired two-tailed Student's *t*-test with Welch's correction.

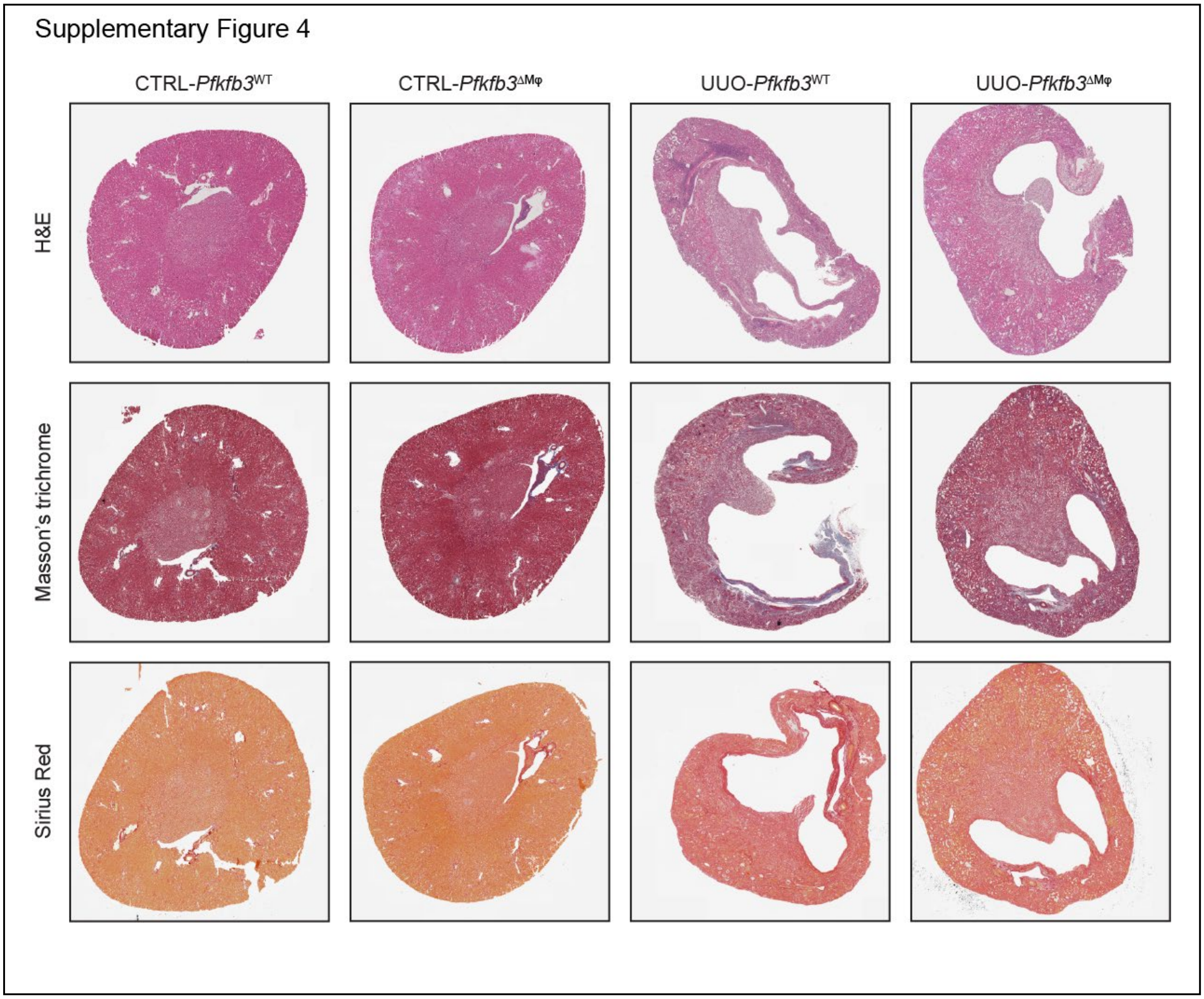

Supplementary Figure 4. The representative images of whole kidney cross section. Control or UUO mouse kidneys were fixed and stained by H&E, Masson's trichrome, or Sirius Red. The whole kidney images were scanned by Zeiss Axioscan7.

Supplementary Figure 5

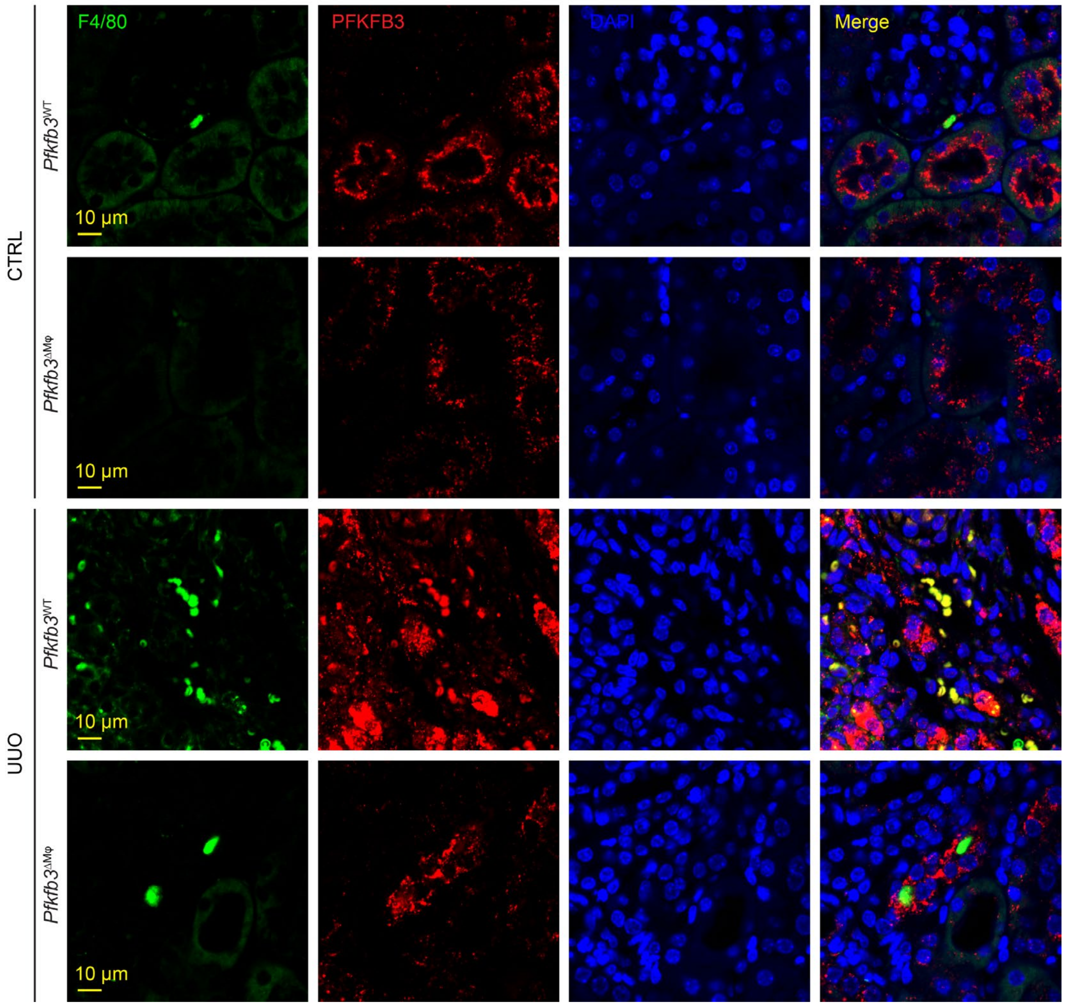

Supplementary Figure 5. The representative images of macrophage and PFKFB3 co-staining. Mouse kidney sections were fixed and stained for F4/80 (green) and PFKFB3 (red). The nuclei were counterstained with DAPI (blue). Images were taken by Zeiss 780 confocal microscope.

Supporting Tables

Supplementary Table 1. Primers used for genotyping of murine strains.

Genotyping	Forward (5´-3´)	Reverse (5´-3´)
Pfkfb3 flox	GGAGTGGCCATTAGGTGGGGTT	CCAGCTTGGGCTACGCATTTAGTT
Lysm Cre	CCCAGAAATGCCAGATTACG	CTTGGGCTGCCAGAATTTCTC
	TTACAGTCGGCCAGGCTGAC	

Supplementary Table 2. Primers used for Quantitative RT-PCR.

Gene (Mouse)	Forward (5´-3´)	Reverse (5´-3´)
18S ribosomal	CTTAGAGGGACAAGTGGCG	ACGCTGAGCCAGTCAGTGTA
Pfkfb3	GATCTGGGTGCCCGTCGATCACCG	CAGTTGAGGTAGCGAGTCAGCTTC
Acta2	ATGCTCCCAGGGCTGTTTTCCCAT	GTGGTGCCAGATCTTTTCCATGT CG
Collagen 1	CCTCAGGGTATTGCTGGACAAC	CAGAAGGACCTTGTTTGCCAGG
Collagen 3	GACCAAAAGGTGATGCTGGACAG	CAAGACCTCGTGCTCCAGTTAG
Mmp2	CGGAAAGTGGAATCCTTGCAGG	AGCAGTGAGGTCAGGCTTGGAA
Mmp9	TTGAAGTCTCAGAAGGTGGAT	GCAGGAGGTCGTAGGTCAC
Tgfb	TTGTTGCCCTCCTACAGACTGG	GTAAAGAGGGCGAAGGCAGCAA
Retnla	CCCTGCTGGGATGACTGCTA	TCCACTCTGGATCTCCCAAGA
Arg1	CAGAAGAATGGAAGAGTCAG	CAGATATGCAGGGAGTCACC
Cd206	TCTTTGCCTTTCCCAGTCTCC	TGACACCCAGCGGAATTTC
Il10	GCTATGCTGCCTGCTCTTACT	CCTGCTGATCCTCATGCCA
Mgl2	TTCAAGAATTGGAGGCCACT	CAGACATCGTCATTCCAACG
Il6	GTTCTCTGGGAAATCGTGGA	TGTACTCCAGGTAGCTATGG
Mcp1	GCTACAAGAGGATCACCAGCAG	GTCTGGACCCATTCCTTCTTGG
F4/80	CGTGTTGTTGGTGGCACTGTGA	CCACATCAGTGTTCCAGGAGAC
Tnfa	ACGGCATGGATCTCAAAGAC	AGATAGCAAATCGGCTGACG
Il1b	TGTCTTGGCCGAGGACTAAGG	TGGGCTGGACTGTTTCTAATGC
Nos2	CAGCTGGGCTGTACAAACCTT	CATTGGAAGTGAAGCGTTTCG
Cxcl10	GAGCCTATCCTGCCCACG	GGAGCCCTTTTAGACCTT
Cd80	ACCCCCAACATAACTGAGTCT	TTCCAACCAAGAGAAGCGAGG

Supplementary Table 3. Antibodies used for Western blot and staining analysis.

Target protein	Company	Catalog #	Concentration
PFKFB3	Abcam	ab181861	1:1000
ACTA2	Santa Cruz Biotechnology	sc-32251	1:1000
Fibronectin (FN)	Abcam	ab2413	1:1000
Collagen I (COL1)	Novus	NB600-408	1:1000
Collagen IV (COL IV)	Abcam	ab6586	1:1000
Vimentin	Cell Signaling Technology	5741	1:1000
HIF1 α	R&D Systems	AF1935	1:500
GAPDH	Cell Signaling Technology	2118S	1:1000
β-actin	Santa Cruz Biotechnology	sc-47778	1:1000
Cyclophilin B	Cell Signaling Technology	43603	1:1000
Anti-mouse IgG, HRP-linked Antibody	Cell Signaling Technology	7076S	1:2000
Anti-rabbit IgG, HRP-linked antibody	Cell Signaling Technology	7074S	1:2000
mouse anti-goat IgG-HRP	Santa Cruz Biotechnology	sc-2354	1:2000
F4/80	Abcam	ab6640	1:100
Arg1	Sigma	AV45673	1:100
IL1β	Abcam	ab9722	1:100
Alexa Fluor 488-conjugated goat anti-mouse secondary antibody	Invitrogen	A11001	1:250
Alexa Fluor 594-conjugated goat anti-rabbit secondary antibody	Invitrogen	A11012	1:250
Alexa Fluor 488-conjugated goat anti-rabbit secondary antibody	Invitrogen	A11008	1:250
Alexa Fluor 594-conjugated goat anti-rat secondary antibody	Invitrogen	A11007	1:250
