# Supplementary material for: Myeloid PFKFB3-mediated glycolysis promotes kidney fibrosis
Source: Front Immunol. 2023 Nov 16;14:1259434. doi: 10.3389/fimmu.2023.1259434 (PMC10687406; doi:10.3389/fimmu.2023.1259434)

Supplementary Figure. Uncropped scans of the Western blots

Figure 3B

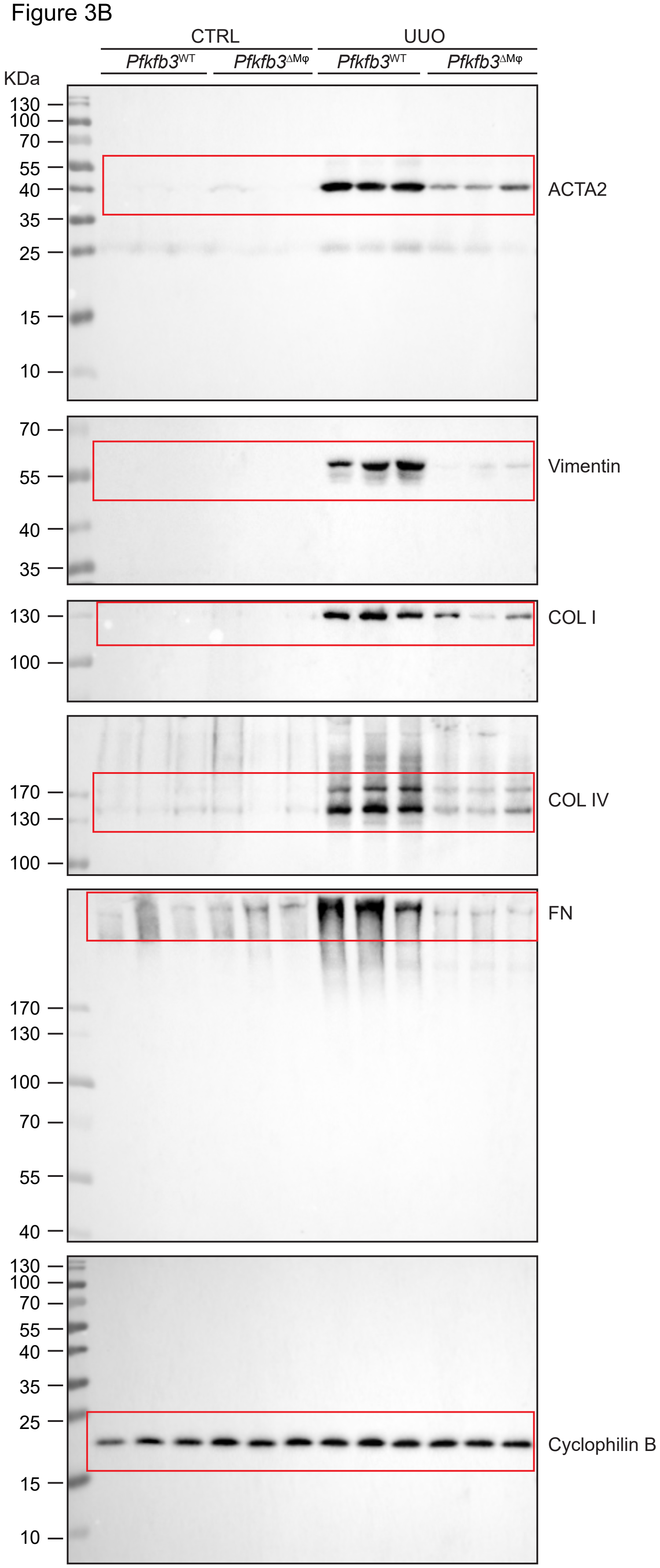

Figure 8B

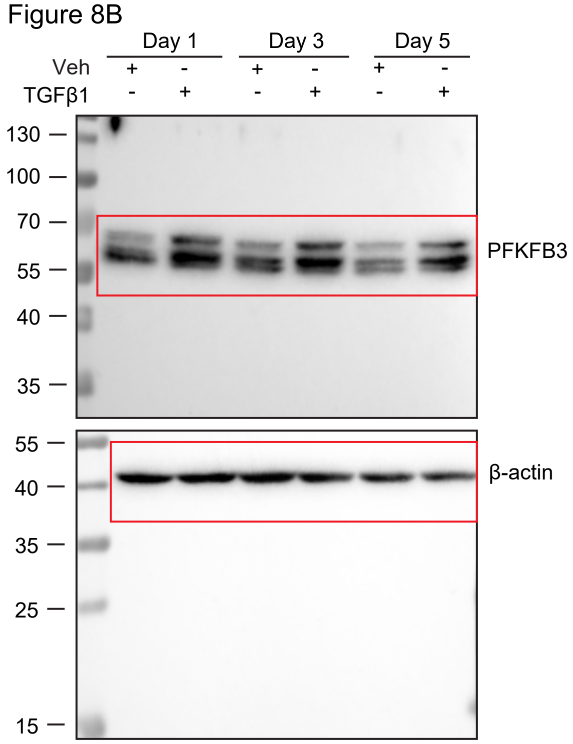

Figure 8G

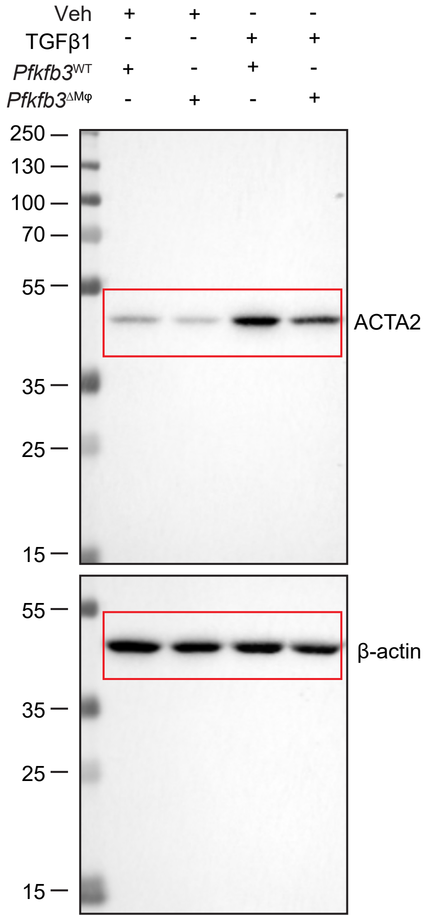

Supplementary Figure. Uncropped scans of the Western blots

Figure 9B

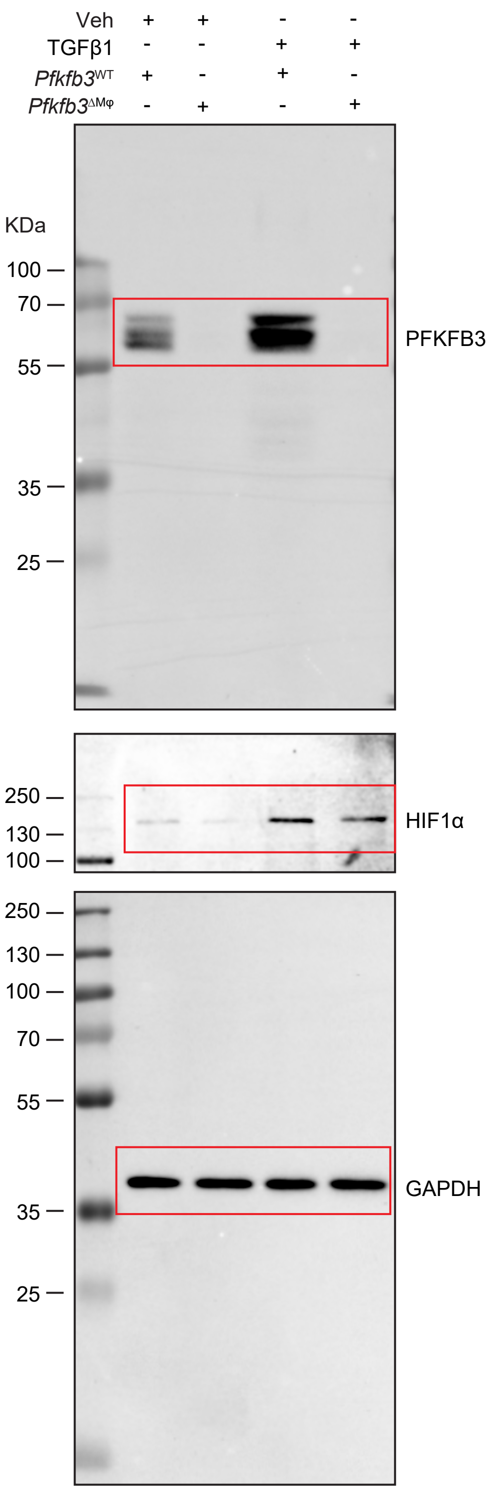

Figure 9C

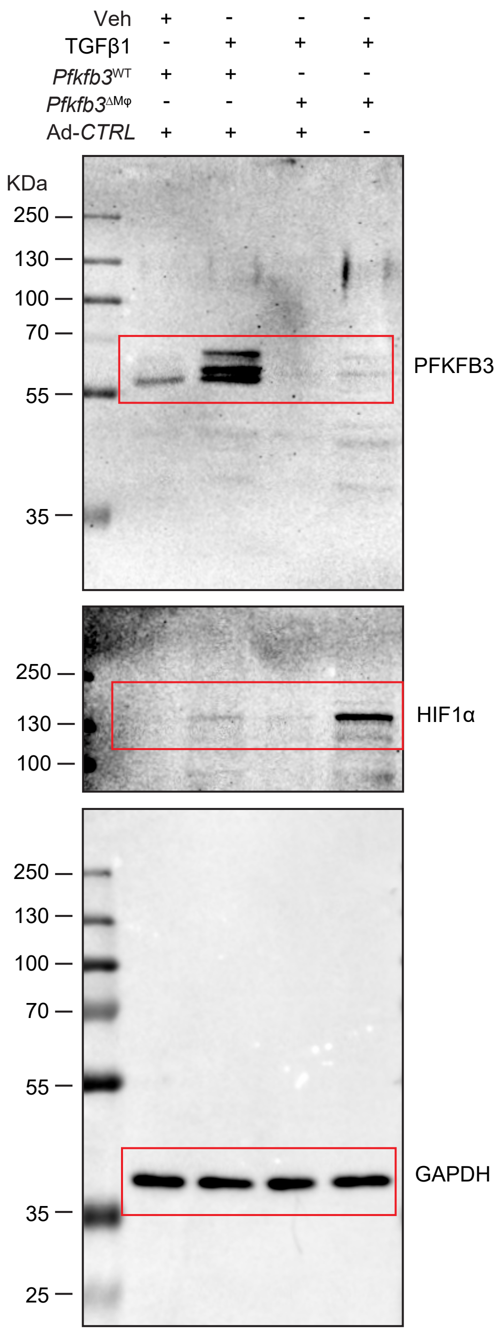

Supplementary Figure 3D

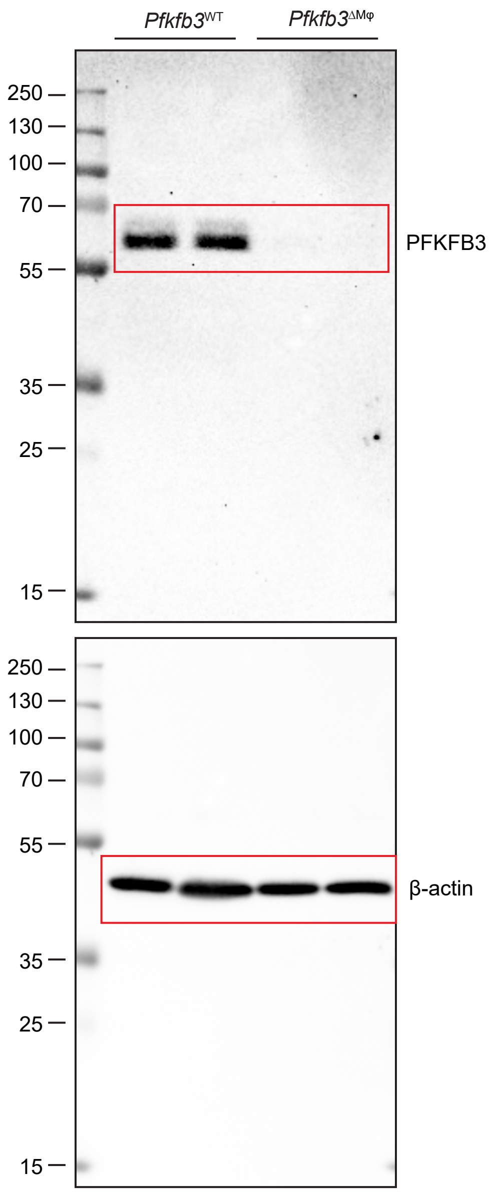

Supplement: Supplementary file 2 [file DataSheet_2.pdf]
